# WNK1 regulates skeletal muscle cell hypertrophy by modulating the nuclear localization and transcriptional activity of FOXO4

**DOI:** 10.1038/s41598-018-27414-0

**Published:** 2018-06-14

**Authors:** Shintaro Mandai, Takayasu Mori, Naohiro Nomura, Taisuke Furusho, Yohei Arai, Hiroaki Kikuchi, Emi Sasaki, Eisei Sohara, Tatemitsu Rai, Shinichi Uchida

**Affiliations:** Department of Nephrology, Graduate School of Medical and Dental Sciences, Tokyo Medical and Dental University, 1-5-45 Yushima, Bunkyo, Tokyo 113-8519 Japan

## Abstract

With-no-lysine (K) (WNK) kinases, which are mutated in the inherited form of hypertension pseudohypoaldosteronism type II, are essential regulators of membrane ion transporters. Here, we report that WNK1 positively regulates skeletal muscle cell hypertrophy via mediating the function of the pro-longevity transcription factor forkhead box protein O4 (FOXO4) independent of the conventional WNK signaling pathway linking SPS/STE20-related proline-alanine–rich kinase (SPAK)/oxidative stress response kinase 1 (OSR1) to downstream effector ion transporters. Small interfering RNA (siRNA)-mediated silencing of WNK1, but not SPAK/OSR1 kinases, induced myotube atrophy and remarkable increases in the mRNA expression of the muscle atrophy ubiquitin ligases MAFbx and MuRF1 in C2C12 mouse skeletal muscle cells. WNK1 silencing also increased FOXO4 nuclear localization, and co-transfection of *Foxo4*-targeted siRNA completely reversed the myotube atrophy and upregulation of atrogene transcription induced by WNK1 silencing. We further illustrated that WNK1 protein abundance in skeletal muscle was increased by chronic voluntary wheel running exercise (hypertrophic stimulus) and markedly decreased by adenine-induced chronic kidney disease (atrophic stimulus) in mice. These findings suggest that WNK1 is involved in the physiological regulation of mammalian skeletal muscle hypertrophy and atrophy via interactions with FOXO4. The WNK1-FOXO4 axis may be a potential therapeutic target in human diseases causing sarcopenia.

## Introduction

Skeletal muscle is the largest tissue in the human body, comprising 40–50% of body mass. This essential tissue also functions as an endocrine organ, regulating metabolic homeostasis. Sarcopenia, defined as a loss of skeletal muscle mass or strength, is a natural part of aging as well as a common co-morbidity after surgery or in various human diseases including cancer, cardiovascular disease, renal failure, and infectious disease^1–5^. Thus, sarcopenia is associated with a major economic burden affecting most older individuals globally given its deleterious effects on functional activities and longevity^6,7^. However, despite the clinical relevance of treating sarcopenia and previous attempts to clarify the mechanism governing muscle hypertrophy/atrophy, efficient countermeasures against sarcopenia other than exercise and nutritional approaches remain to be discovered.

We previously demonstrated that Na^+^-K^+^-2Cl^−^ co-transporter 1 (NKCC1), a member of the SLC12A family cation-coupled chloride transporter family, is an important regulator of mammalian skeletal myogenesis, and loop diuretics, which inhibit the transporter, may clinically underlie the pathogenesis of sarcopenia^8^. The other SLC12A family members NKCC2 and Na^+^-Cl^−^ co-transporter (NCC), which are mutated in the salt-losing nephropathies Bartter syndrome and Gitelman syndrome respectively, are known to physiologically govern salt reabsorption and blood pressure in the kidneys and arteries^9,10^. The primary upstream regulators of these SLC12A transporters are with-no-lysine (K) (WNK) serine/threonine protein kinases^9,10^, including the four mammalian isoforms WNK1, WNK2, WNK3, and WNK4. WNK kinases phosphorylate and activate SLC12A transporters via the intermediate kinases SPS/STE20-related proline-alanine–rich kinase (SPAK) and the SPAK homolog oxidative stress response kinase 1 (OSR1), constituting the WNK signaling pathway^9,10^. Genetic mutations in either WNK1 or WNK4 cause the autosomal dominant hereditary hypertensive disease pseudohypoaldosteronism type II (PHAII) via NCC activation, and the phenotype of PHAII is opposite of that of Gitelman syndrome, which is caused by loss of function of NCC^11,12^. Recently, the roles of WNK kinases in various extrarenal tissues, such as the brain, bone, adipocytes, and immune cells, have been increasingly explored^13–16^. In particular, WNK1 is ubiquitously expressed in mammalian tissues, in which it was demonstrated to regulate essential cellular processes including cell proliferation, mitosis, and autophagy^17–19^. However, the function of WNK kinases in skeletal muscle is unknown.

In this study, we investigated the role of WNK kinases in skeletal muscle hypertrophy with a specific focus on WNK1 because of its abundant expression in mammalian skeletal muscles^20^ and a recent report of a family with limb-girdle muscular dystrophy caused by a *WNK1* mutation^21^. We demonstrated that WNK1 positively regulates muscle hypertrophy in C2C12 mouse skeletal muscle cells. This effect was attributable to a previously unrecognized link between WNK1 and the pro-longevity transcription factor forkhead box protein O4 (FOXO4), which regulates lifespan extension, tumor suppression, and energy metabolism^22^.

## Results

### WNK1 protein expression is upregulated during C2C12 myoblast differentiation

To clarify whether WNK1 is expressed in mouse C2C12 skeletal muscle cells and muscle tissue, we examined WNK1–4 mRNA levels in C2C12 cells and various tissues via reverse transcription (RT)-PCR, as well as WNK1 protein levels in differentiating C2C12 cells via Western blotting.

**Figure 1:**
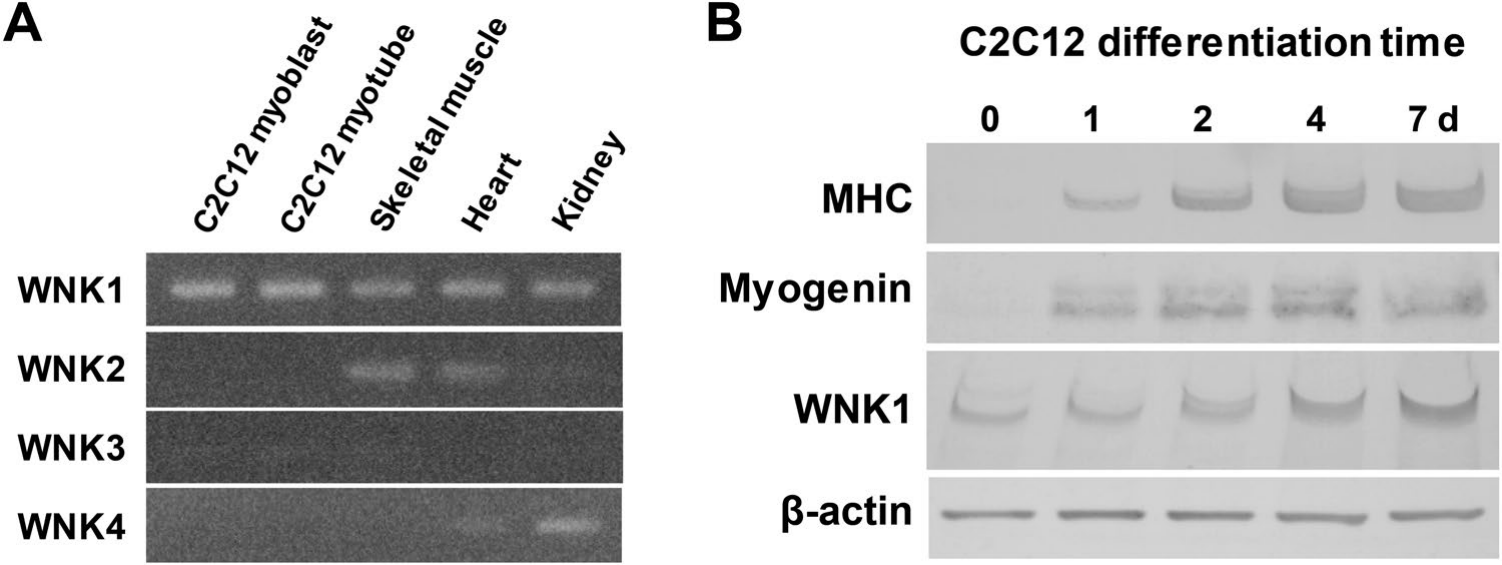
mRNA and protein expression levels of WNK1 in mouse skeletal muscle and differentiating C2C12 mouse skeletal muscle cells. (**A**) Reverse transcription polymerase chain reaction for four mammalian WNK isoforms WNK1, WNK2, WNK3, and WNK4 in C2C12 pre-differentiation myoblasts, differentiated myotube, mouse quadriceps, heart, and kidney. Only WNK1 was abundantly expressed in both C2C12 cells and skeletal muscle tissue. (**B**) Immunoblots for WNK1 and myogenic markers in differentiating C2C12 cells. WNK1 expression was increasingly upregulated during differentiation in parallel with the increasing expression of the myogenic markers myogenin and myosin heavy chain. MHC, myosin heavy chain; WNK, with-no-lysine (K). Full-length blots/gels are presented in Supplementary Fig. 7.

WNK1 and WNK2 mRNA was expressed in mouse skeletal muscle (Fig. 1A). A previous study showed the very low abundance of WNK2 protein expression in mouse skeletal muscle^23^. Only WNK1 was abundantly expressed in both C2C12 cells and skeletal muscle tissue, suggesting that WNK1 is the major WNK isoform in skeletal muscle. WNK1 was also highly expressed in the heart, another striated muscle tissue (Fig. 1A). WNK1 transcripts were expressed in pre-differentiation C2C12 myoblasts. Of note, WNK1 expression was increasingly upregulated after switching to differentiation medium (DM), in parallel with the increasing expression of myogenin and myosin heavy chain (MHC), which are muscle-specific proteins and myogenic markers (Fig. 1B). These findings suggest that WNK1 may play a substantial role in muscle hypertrophy or myogenesis.

### WNK1 silencing induced myotube atrophy and increased atrogene expression

**Figure 2:**
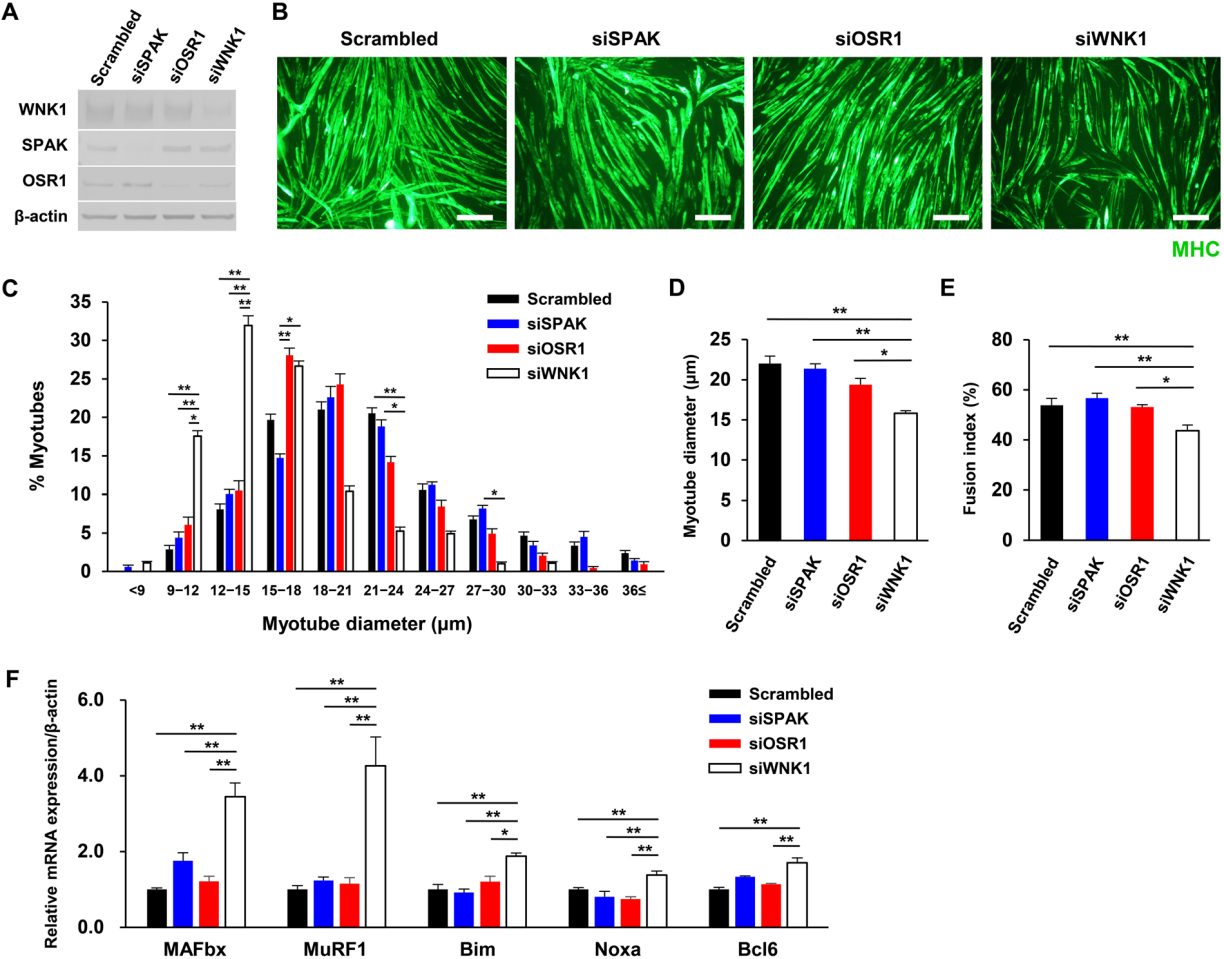
Small interfering RNA (siRNA)-mediated silencing of WNK1, but not SPAK/OSR1 kinases, induced myotube atrophy and increased atrogene expression. (**A**) Immunoblots showing the siRNA knockdown efficiency of WNK1, SPAK, or OSR1 in C2C12 cells. (**B**) After C2C12 cells were pre-treated with siRNA, the cells were incubated in differentiation medium for 4 days, and immunofluorescence study was performed with a myosin heavy chain (MHC) antibody. Scale bar, 200 μm. (**C**) Histograms showing proportions of myotubes according to the diameters. The proportions of atrophic myotubes were markedly higher in the WNK1-silenced group (*n* = 5 or 6 per experimental group). (**D**) The mean myotube diameter value in the WNK1-silenced group was lower than that in the control group (*n* = 5 or 6 per experimental group). (**E**) The fusion index represents the percentage of multinucleated MHC-positive myotubes per vision field after 96 h of differentiation (*n* = 5 or 6 per experimental group). *Wnk1*-targeted siRNA suppressed the myogenic fusion of myoblasts into myotubes. (**F**) Quantification of MAFbx, MuRF1, Bim, Noxa, and bcl6 in siRNA-treated C2C12 cells by real-time polymerase chain reaction analysis (*n* = 4 per experimental group). mRNA levels were normalized against those of *β-actin*. Values are presented as the mean ± standard error of the mean. *P < 0.05; **P < 0.01. Small interfering RNA, siRNA; WNK, with-no-lysine (K); SPAK, SPS/STE20-related proline-alanine–rich kinase; OSR1, oxidative stress responsive 1; MHC, myosin heavy chain; MAFbx, muscle atrophy F-box; MuRF1, Muscle RING-finger protein-1. Full-length blots are presented in Supplementary Fig. 8.

To determine whether WNK1 modulates muscle cell hypertrophy or myogenic differentiation, we measured myotube diameter and the myoblast fusion index^8^ after small interfering RNA (siRNA)-mediated silencing of WNK1, SPAK, or OSR1 (Fig. 2A) using immunofluorescence for MHC in differentiated C2C12 myotubes 4 days after switching the culture medium to DM.

As shown in Fig. 2B, the number and diameter of MHC-positive multinucleated mature myotubes after 96 h of differentiation were reduced when WNK1 was silenced. Histograms of myotube diameters revealed markedly higher proportions of atrophic myotubes following WNK1 silencing compared to the control findings, whereas the siSPAK and siOSR1 groups displayed similar distributions as the control group (Fig. 2C). The mean myotube diameter value in the WNK1-silenced group was lower than that in the control group (Fig. 2D). The calculated fusion index^8^, which represents the percentage of multinucleated MHC-positive myotubes, was also lower in the WNK1-silenced group, suggesting that the myogenic fusion of myoblasts into myotubes was impaired by WNK1 silencing (Fig. 2E).

One of the primary mechanisms underlying mammalian skeletal muscle atrophy is activation of the ubiquitin-proteasome system by the muscle-specific E3 ubiquitin ligases muscle atrophy F-box (MAFbx) and muscle RING-finger protein-1 (MuRF1), which are known as ‘atrogenes’ that promote muscle protein degradation^24–26^. To further examine if changes in atrogene expression are involved in myotube atrophy induced by WNK1 silencing, we performed quantitative RT-PCR to examine MAFbx and MuRF1 mRNA expression in C2C12 cells.

The results illustrated that MAFbx and MuRF1 mRNA levels were markedly increased by WNK1 silencing (Fig. 2F), but not SPAK or OSR1 silencing. Transcription of these atrogenes is predominantly regulated by FOXOs^27^. Thus, other factors downstream of FOXOs, including Bim, Noxa, and bcl6, were additionally evaluated. The results indicated that WNK1 silencing also led to significant increases in Bim, Noxa, and bcl6 mRNA expression compared to the findings in control cells. Furthermore, the changes induced by WNK1 silencing were replicated in H9C2 rat myocytes (Fig. S1). These findings suggest that WNK1 plays an essential role in skeletal muscle cell hypertrophy via the transcriptional regulation of atrogenes, presumably by modulating the function of FOXOs but not SPAK/OSR1.

We further evaluated the effect of human WNK1 overexpression on myotube diameter and atrogene expression in C2C12 cells. As shown in Fig. S2A, WNK1 overexpression did not increase phosphorylation of its substrates SPAK/OSR1, suggesting that the basal expression of endogenous WNK1 is abundant and its kinase activity is saturated in C2C12 cells. This finding presumably explains that WNK1 overexpression did not modulate myotube diameter and atrogene expression, either (Fig. S2B–E).

### WNK1 silencing increased FOXO4 nuclear localization in C2C12 cells

Three FOXO isoforms, namely FOXO1, FOXO3, and FOXO4, are expressed in mammalian skeletal muscle^28^. To clarify which FOXO isoform functions as the effector in the WNK1-mediated regulation of atrogene transcription and myotube hypertrophy in C2C12 cells, the nuclear and cytoplasmic protein levels of FOXO isoforms were evaluated by immunoblotting after separating nuclear/cytoplasmic extracts in WNK1-silenced C2C12 cells.

**Figure 3:**
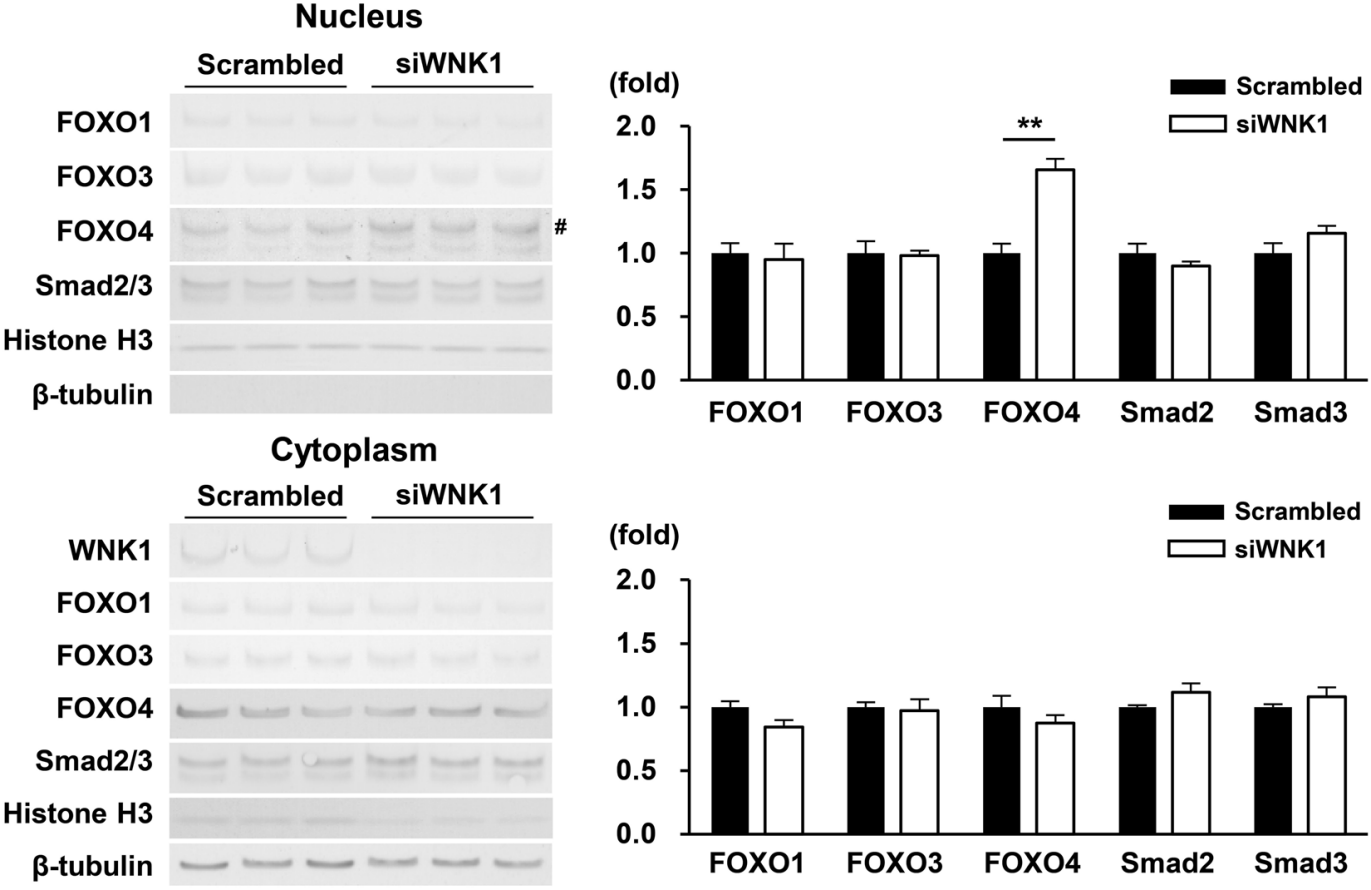
WNK1 silencing increased FOXO4 nuclear localization in C2C12 cells. Immunoblots (left) and densitometric analyses (right) of three FOXO isoforms, namely FOXO1, FOXO3, and FOXO4, as well as Smad2/3 after separating nuclear (upper)/cytoplasmic (lower) extracts in WNK1-silenced C2C12 cells. FOXO4 levels were selectively increased in the nuclei of C2C12 cells treated with WNK1-targeted siRNA. The hash (#) shows FOXO4. Values are presented as the mean ± standard error of the mean (*n* = 4 or 7 per experimental group). *P < 0.05 versus the control cells. Full-length blots are presented in Supplementary Fig. 9.

The protein bands of nuclear β-tubulin and cytoplasmic Histone H3 were faint, suggesting the efficient separation of the nuclear/cytoplasmic extracts of the cells (Fig. 3). The nuclear abundance of FOXO4 was significantly increased by WNK1 silencing, whereas FOXO1 and FOXO3 levels were not altered. Regarding other transcription factors regulating atrogene transcription such as Smad2, Smad3, Nuclear factor-κB p65, and p38 mitogen-activated protein kinase^29^, their nuclear and cytoplasmic localization was not altered (Figs 3 and S3). These findings suggest that FOXO4 levels were selectively increased in the nuclei of C2C12 cells treated with *Wnk1*-targeted siRNA, which may explain the upregulation of atrogene transcription and myotube atrophy in these cells.

### FOXO4 silencing rescued WNK1 silencing induced muscle cell atrophy and atrogene upregulation

**Figure 4:**
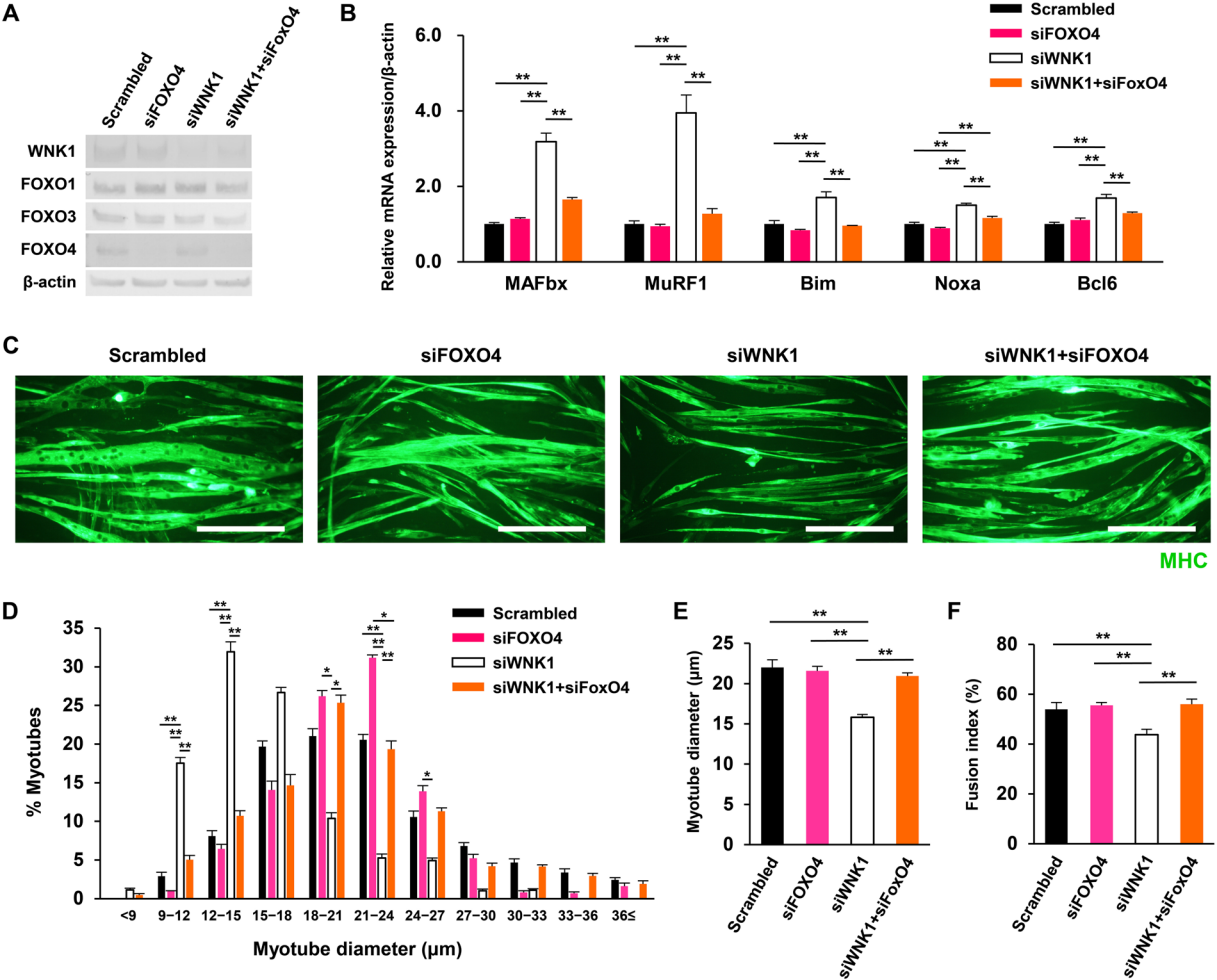
FOXO4 silencing rescued WNK1 silencing induced muscle cell atrophy and atrogene upregulation. (**A**) Immunoblots showing the siRNA knockdown efficiencies of WNK1 and FOXO4 in C2C12 cells. (**B**) Quantification of MAFbx, MuRF1, Bim, Noxa, and bcl6 in siRNA-treated C2C12 cells by real-time polymerase chain reaction analysis (*n* = 4 or 6 per experimental group). The upregulation of these transcripts induced by WNK1 silencing was cancelled upon co-transfection of *Foxo4*-targeted siRNA. mRNA levels were normalized against those of *β-actin*. (**C**) Immunofluorescence with a myosin heavy chain (MHC) antibody in C2C12 cells that were pre-treated with siRNA and incubated in differentiation medium for 4 days. Myotube atrophy induced by WNK1 silencing was substantially rescued by co-silencing of FOXO4. Scale bar, 200 μm. (**D**,**E**) Histograms showing proportions of myotubes according to the diameters. The shift to a higher proportion of atrophic myotubes upon WNK1 silencing was completely reversed by FOXO4 silencing **(D)**, as was the reduction of the mean myotube diameter (**E**) (*n* = 5 or 6 per experimental group). (**F**) The decrease in the fusion index, indicating impaired myogenesis, induced by WNK1 silencing was also cancelled by FOXO4 silencing (*n* = 5 or 6 per experimental group). Values are presented as the mean ± standard error of the mean. *P < 0.05; **P < 0.01. Small interfering RNA, siRNA; WNK, with-no-lysine (K); FOXO4, forkhead box protein O4; MHC, myosin heavy chain; MAFbx, muscle atrophy F-box; MuRF1, Muscle RING-finger protein-1. Full-length blots are presented in Supplementary Fig. 10.

We next validated whether the altered nuclear/cytoplasmic localization and transcriptional activity of FOXO4 was responsible for C2C12 muscle cell atrophy induced by WNK1 silencing. We evaluated myotube diameter and the myoblast fusion index using immunofluorescence for MHC, as well as the mRNA expression of atrogenes and factors downstream of FOXO4 (Fig. 2F) using quantitative RT-PCR, after co-transfection of *Foxo4*- and *Wnk1*-targeted siRNA in C2C12 cells (Fig. 4A).

First, as shown in Fig. 4B, the upregulation of MAFb, MuRF1, Bim, Noxa, and bcl6 transcripts induced by WNK1 silencing was cancelled upon co-transfection of *Foxo4*-targeted siRNA. Second, in line with these findings, myotube atrophy induced by WNK silencing was substantially rescued by co-silencing of FOXO4. In the analyses of myotube diameters, the shift to a higher proportion of atrophic myotubes upon WNK1 silencing was completely reversed by FOXO4 silencing (Fig. 4C), as was the reduction of the mean myotube diameter (Fig. 4D). Moreover, the decrease in the fusion index, indicating impaired myogenesis, induced by WNK1 silencing was also cancelled by FOXO4 silencing. These findings confirmed that WNK1 regulates mouse skeletal muscle cell hypertrophy and myogenic differentiation by modulating the nuclear localization and transcriptional activity of FOXO4.

### WNK1 modulates FOXO4 phosphorylation

To determine whether WNK1 is involved in the phosphorylation of FOXO4, which potentially modulates its nuclear localization and transcriptional activity, phosphorylation of endogenous FOXO4 at three conserved AKT phosphorylation sites^30–32^ was evaluated in C2C12 cells via immunoblotting.

**Figure 5:**
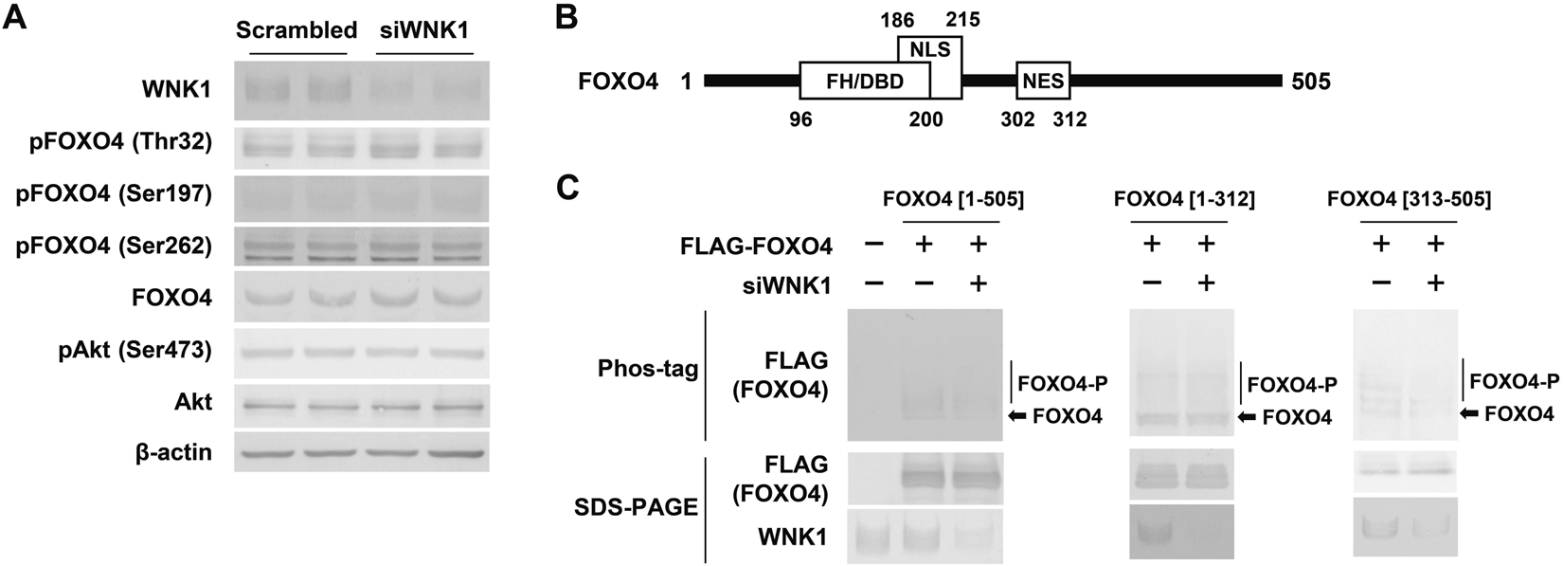
WNK1 silencing reduced FOXO4 phosphorylation at different sites from those phosphorylated by Akt. (**A**) Immunoblots showing that FOXO4 phosphorylation at T32, S197, or S262, which promotes FOXO4 nuclear exclusion and inactivates the protein, was not decreased by WNK1 silencing in C2C12 cells. (**B**) Domain structure of human FOXO4 protein. (**C**) Phos-tag SDS-PAGE revealed that the abundance of upper smear bands representing phosphorylated full-length FOXO4 [1–505] was decreased in WNK1-silenced HEK 293 T cells compared with that in control cells (Fig. S3B,C). The abundance of phosphorylated bands of FOXO4 [1–312] was unchanged in WNK1-silenced cells, whereas that of FOXO4 [313–505] was decreased. FH/DBD, forkhead box/DNA-binding domain; NLS, nuclear localization sequence, NLS; NES, nuclear export sequence; WNK1, with-no-lysine (K); FOXO4, Forkhead box protein O4. Full-length blots are presented in Supplementary Fig. 11.

As shown in Fig. 5A, FOXO4 phosphorylation at T32, S197, or S262, which promotes FOXO4 nuclear exclusion and inactivates the protein^30–32^, was not decreased by WNK1 silencing. Akt phosphorylation at S473, which triggers its activity, was also not changed by WNK1 silencing.

We further validated whether other FOXO4 phosphorylation sites are associated with WNK1, using phos-tag SDS-PAGE, which detects phosphorylation at serine, threonine, or tyrosine residues^33^. Given that phosphorylation of endogenous FOXO4 in C2C12 cells could not be detected with the anti-FOXO4 antibody using phos-tag SDS-PAGE, that of FLAG-tagged human FOXO4 was evaluated in WNK1-silenced and control HEK293T cells.

Phos-tag SDS-PAGE revealed that the abundance of upper smear bands representing phosphorylated full-length FOXO4 [1–505] was decreased in WNK1-silenced cells compared with that in control cells (Fig. 5B,C). In addition, when two truncated FOXO4 fragments, FOXO4 [1–312] and FOXO4 [313–505], were analyzed, the abundance of phosphorylated bands of FOXO4 [1–312] was unchanged in WNK1-silenced cells, whereas that of FOXO4 [313–505] was decreased. These results suggest that WNK1 is involved in FOXO4 phosphorylation at serine, threonine, or tyrosine residues located in the [313–505] domain, and phosphorylation sites other than T32, S197, and S262 could also facilitate FOXO4 nuclear export and inactivation of its transcriptional activity.

**Figure 6:**
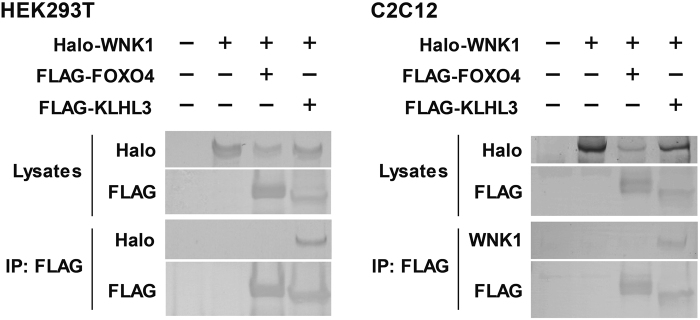
WNK1 was not co-immunoprecipitated with FOXO4. Halo-tagged human WNK1 was co-immunoprecipitated with FLAG-tagged human KLHL3, but not with human FOXO4, in HEK293T (left) and C2C12 cells (right). WNK1, with-no-lysine (K); FOXO4, Forkhead box protein O4; kelch like family member 3, KLHL3. Full-length blots are presented in Supplementary Fig. 12.

Moreover, we performed co-immunoprecipitation in HEK293T and C2C12 cells. As shown in Fig. 6, Halo-tagged human WNK1 was co-immunoprecipitated with FLAG-tagged human KLHL3, but not with human FOXO4.

### Effects of chronic exercise and renal failure on WNK1 levels

To investigate whether WNK1 is physiologically involved in the mechanism underlying *in vivo* exercise-induced skeletal muscle hypertrophy or muscle atrophy related with common human diseases, we examined the effect of 6 weeks of voluntary wheel running exercise^8^ or adenine-induced chronic kidney disease (CKD)^29^ on WNK1 protein levels in mouse skeletal muscle.

**Figure 7:**
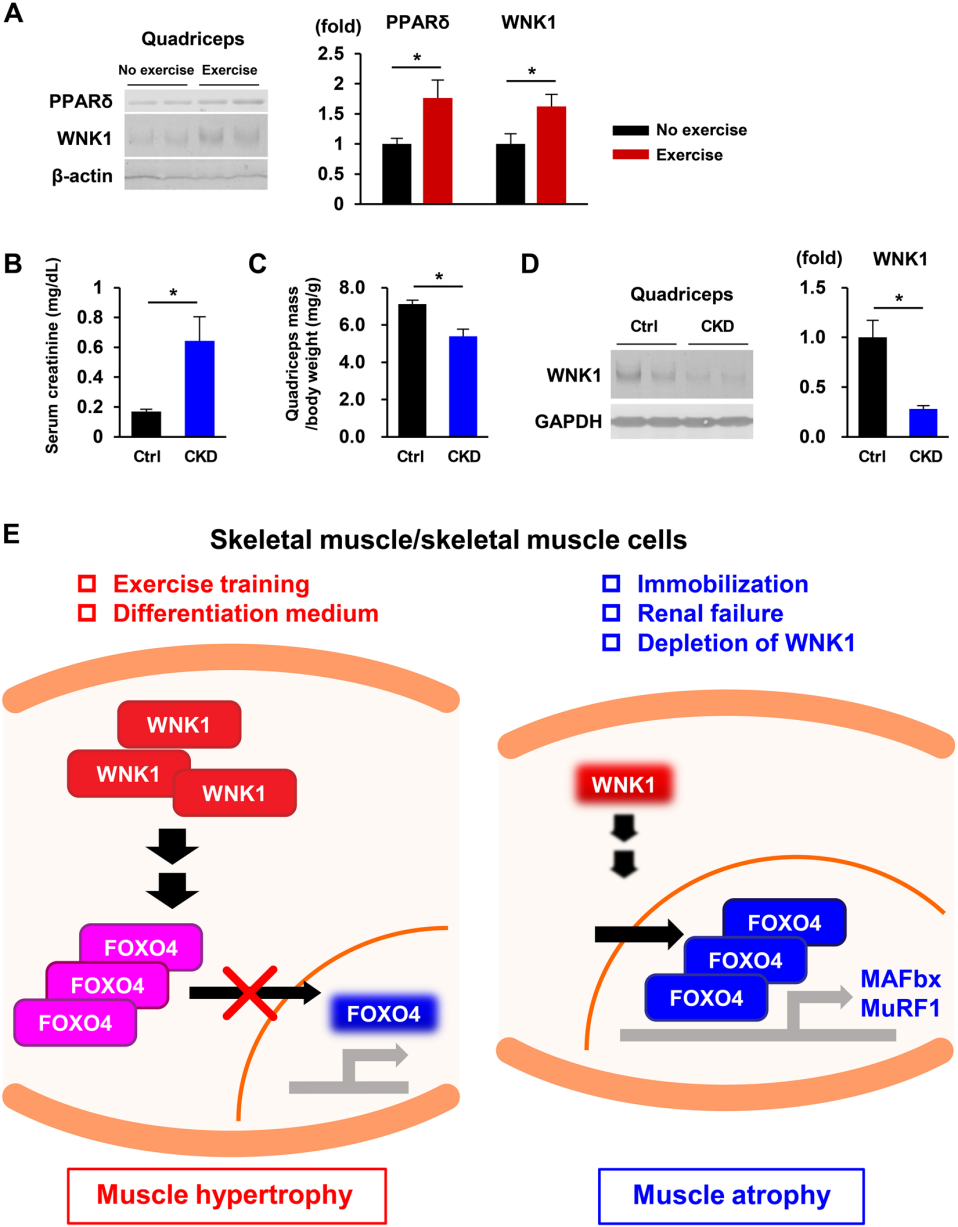
WNK1 is involved in the physiologic regulation of mammalian skeletal muscle hypertrophy and atrophy in response to environmental or pathophysiological stresses. (**A**) Representative immunoblots (left) and densitometric analysis (right) of PPARδ and WNK1 in mouse quadriceps after 6 weeks of voluntary wheel running exercise (*n* = 5 per experimental group). (**B**,**C**) Four weeks of adenine-containing diet feeding resulted in significant increases in serum creatinine levels (**B**) and decreases in body weight-correct quadriceps muscle mass (**C**), indicating the successful induction of chronic kidney disease (CKD) and sarcopenia (*n* = 3 per experimental group). (**D**) Representative immunoblots (left) and densitometric analysis (right) of muscle lysates revealed a marked decrease of WNK1 levels in the CKD mice (*n* = 3 per experimental group). Values are presented as the mean ± standard error of the mean. *P < 0.05 versus the control mice. (**E**) Models illustrating the regulation of mammalian skeletal muscle hypertrophy and atrophy by WNK1. Muscle hypertrophic stimuli upregulate WNK1 protein levels, resulting in the nuclear exclusion and decreased transcriptional activity of FOXO4 (left). By contrast, atrophic stimuli decrease WNK1 expression and increase the nuclear localization and transcriptional activity of FOXO4 together with increased atrogene transcription, leading to muscle protein degradation and ultimately muscle atrophy (right). PPAR, peroxisome proliferator-activated receptor; WNK, with-no-lysine (K); FOXO4, forkhead box protein O4; chronic kidney disease, CKD. Full-length blots are presented in Supplementary Fig. 13.

As shown in Fig. 7A, chronic exercise training upregulated WNK1 protein levels together with those of the well-known exercise-induced marker peroxisome proliferator-activated receptor (PPAR) δ^8^ in mouse quadriceps. By contrast, 4 weeks of adenine-containing diet feeding resulted in significant increases in serum creatinine levels and decreases in body weight-correct quadriceps muscle mass, indicating the successful induction of CKD and sarcopenia (Fig. 7B,C). Immunoblotting of mouse muscle lysates revealed a marked decrease of WNK1 levels in adenine-treated mice. These findings suggest that WNK1 protein levels are physiologically modulated by various environmental or pathophysiological stresses, and these changes have a pivotal role in mammalian skeletal muscle hypertrophy *in vivo* (Fig. 7D).

### A WNK kinase inhibitor increased atrogene expression in mouse skeletal muscle

Given that WNK1 is the major WNK isoform in skeletal muscle and only WNK1 isoform is expressed in C2C12 cells (Fig. 1)^23^, to further clarify the role of WNK1 in the regulation of muscle hypertrophy *in vivo*, we evaluated the effect of the orally bioavailable pan-WNK kinase inhibitor called WNK463^34^ on atrogene expression in mouse skeletal muscle and C2C12 cells.

We initially revealed that WNK463 induced a dose-dependent decrease in C2C12 differentiated myotube diameter (Fig. S4A,B). Moreover, 10 μM WNK463 time-dependently upregulated transcription of MAFbx and MuRF1 (Fig. S4C). Administration of WNK463 by oral gavage (10 mg/kg) was previously shown to substantially inhibit WNK kinase activity *in vivo* and decrease phosphorylation of substrates SPAK/OSR1 in mouse kidneys^34^. We confirmed the decrease of phosphorylated SPAK in kidneys at 1.5 h after WNK463 administration (Fig. S4D), suggesting the sufficient inhibition of WNK kinase activity *in vivo*. WNK463 further increased mRNA expressions of MAFbx and MuRF1 at 6 h after administration (Fig. S4E). These findings suggest that WNK1 plays a role in regulating skeletal muscle hypertrophy dependent of its kinase activity *in vivo*.

## Discussion

The present study demonstrated that WNK1 kinase, the protein abundance of which is increased during myoblast differentiation into myotubes, positively controls myotube hypertrophy. This function of WNK1 in skeletal muscle cells was attributable to modulation of the nuclear localization and transcriptional activity of FOXO4 together with increased atrogene expression independent of SPAK and OSR1 function^9,10^. These results demonstrate a novel interaction between WNK1 and the pro-longevity factor FOXO4. We also revealed that WNK1 protein expression is upregulated by chronic exercise training (muscle hypertrophic stimulus) and downregulated by CKD (atrophic stimulus) in mouse skeletal muscle. These findings suggest that WNK1 is a physiological regulator of mammalian skeletal muscle hypertrophy.

WNK1 causes hypertension and hyperkalemia when overexpressed in the kidneys^9,10^ and embryonically lethal cardiovascular defects when homozygously deleted in animals^35,36^. In recent decades, a number of studies employing functional analyses of the human mutations causing PHAII revealed that the phenotype associated with WNK1 overexpression is based on activation of the WNK-SPAK/OSR1-SLC12A signaling pathway in renal tubules^9,10,37–39^. Conversely, that associated with homozygous WNK1 deletion is linked with the multifactorial functions of WNK1, and the mechanisms are not fully understood. In particular, the embryonic lethality caused by homozygous WNK1 deletion cannot be explained by disturbance of the WNK-SPAK/OSR1-SLC12A pathway, given that *Wnk4*^*−/−*^ mice do not exhibit embryonic lethality or a shortened life span^15,39^. These findings indicate that functions of WNK1 related to fundamental biological processes independent of SPAK/OSR1 remain unrevealed. In fact, recent studies reported that WNK1 is essential for cell proliferation, mitosis, and autophagy^16–19^.

The present study is the first to investigate the pivotal role of WNK1 in skeletal muscle. siRNA-mediated silencing of WNK1, but not SPAK and OSR1, caused myotube atrophy in C2C12 cells (Fig. 2). In our previous study, we reported that NKCC1, a substrate of SPAK/OSR1, promotes myogenesis^8^, and our initial assumption was that NKCC1 might also be a downstream effector of WNK1 in skeletal muscle, similar to the findings in the kidneys or arteries^9,10^. Therefore, the findings of this study were completely unexpected. In particular, of interest is that myotube atrophy induced by WNK1 silencing was accompanied by markedly increased transcription of MAFbx and MuRF1 (Fig. 2). MAFbx and MuRF1 are known as atrogenes that promote muscle protein degradation via the ubiquitin-proteasome pathway and as the primary effectors of muscle atrophy in various human chronic diseases, aging, and disuse conditions^24–26^. Atrogene expression levels are transcriptionally regulated, and the predominant transcription factors are FOXO family members including FOXO1, FOXO3, and FOXO4 in mammalian skeletal muscle^28^. Accordingly, other FOXO-mediated transcripts including Bim, bcl6, and Noxa were also upregulated by WNK1 silencing. These WNK1-mediated alterations in FOXO-regulated transcripts were apparently replicated in H9C2 rat myocytes, which are also mammalian striated muscle cells (Fig. S1).

We further determined that the increase in FOXO4 nuclear localization among FOXOs was responsible for the upregulated transcription of atrogenes upon WNK1 silencing (Fig. 3). Co-transfection of *Foxo4*-targeted siRNA completely rescued the myotube atrophy and increased atrogene transcription induced by WNK1 silencing (Fig. 4), confirming the previously unrecognized link between WNK1 and FOXO4 in skeletal muscle. Previous *in vitro* and in *vivo* studies investigating the role of FOXOs in skeletal muscle predominantly focused on FOXO1 and FOXO3 because of their higher expression in mammalian skeletal muscle than FOXO4^28^. Thus, the specific function of FOXO4 on myogenic differentiation and hypertrophy has not been fully understood, in contrast to those of FOXO1 and FOXO3^40,41^. Our study demonstrated the role of FOXO4 in myogenesis and muscle cell hypertrophy in relation to the novel upstream regulator WNK1.

The mechanism underlying the WNK1-mediated modulation of FOXO4 nuclear/cytoplasmic localization and transcriptional activity remains to be clarified. Phosphorylation is among the major post-transcriptional modifications that modulate the transcriptional activity of FOXO4^22^. Phos-tag SDS-PAGE revealed that WNK1 is involved in FOXO4 phosphorylation in the [313–505] domain, which might promote nuclear exclusion of FOXO4 and inactivate the protein. However, WNK1 was not co-immunoprecipitated with FOXO4 (Fig. 6). Additional intermediate or adaptor proteins might be involved in the WNK1-FOXO4 axis. Further examination is necessary to confirm the phosphorylation of FOXO4 by WNK1 and its relationship with the transcriptional activity of FOXO4.

In our mouse experiments, voluntary wheel running exercise, which induces muscle hypertrophy^8^, increased WNK1 protein levels in muscle (Fig. 7). By contrast, CKD had the opposite effect. Previous studies demonstrated that exercise training efficiently attenuated CKD-induced muscle atrophy by suppressing MAFbx and MuRF1 transcription^42,43^. Of interest is that a pan-WNK kinase inhibitor WNK463 increased atrogene transcription in mouse skeletal muscle and C2C12 cells (Fig. S4). We showed that WNK1 is the major WNK isoform in mammalian skeletal muscle, based on the findings in the present study (Figs 1 and S1) and previous report^23^. These findings suggest that WNK1 kinase activity is essential in regulating skeletal muscle hypertrophy and might be related with the pathogenesis underlying human diseases causing sarcopenia, although further examinations upon specific silencing or deletion of WNK1 in skeletal muscle are needed for the confirmation.

The modulation of WNK1 protein levels in response to chronic exercise or CKD was not attributable to that of WNK1 transcript levels (Fig. S5A,B), presumably suggesting the involvement of WNK1 protein degradation. WNK1 and WNK4 are substrates of kelch like family member 3 (KLHL3)-Cullin3 E3 ligase^38,44^, and WNK1 protein expression is predominantly regulated by KLHL3 and Cullin3 in the kidneys^9^. However, these proteins are not involved in WNK1 degradation in mouse skeletal muscle^45^ or C2C12 cells, based on the finding that Cullin3 silencing did not alter WNK1 expression levels (Fig. S5C). Another degradation system may physiologically regulate muscle WNK1 abundance. Zhang YJ *et al*.^19^ recently showed that WNK1 is essential for hypotonic-induced proliferation of vascular smooth muscle cells, by mediating cell cycle transition from G_0_/G_1_ to S phase through phosphatidylinositol 3-kinase (PI3K)-Akt signaling pathway and modulation of WNK1 phosphorylation^46^. The PI3K/Akt pathway was not involved in myotube atrophy induced by WNK1 silencing in skeletal muscle cells (Fig. 5A). However, various stimuli such as exercise or insulin might modulate this pathway and WNK1 phosphorylation. Additional experiments are needed for understanding the modulation of WNK1 abundance and phosphorylation under physiological stimuli.

In conclusion, WNK1 positively regulates skeletal muscle cell hypertrophy by modulating the nuclear localization and transcriptional activity of FOXO4. The alteration of mouse skeletal muscle WNK1 protein levels in response to chronic exercise training (muscle hypertrophic stimulus) or CKD (atrophic stimulus) indicates a physiological function of WNK1 in mammalian skeletal muscle hypertrophy. The WNK1-FOXO4 axis may be a novel therapeutic target for treating sarcopenia.

## Methods

### Cell cultures and transfection

C2C12 mouse skeletal muscle cells, H9C2 rat myocytes, and HEK293T cells were cultured in DMEM (Invitrogen, Carlsbad, CA, USA) supplemented with 10% fetal bovine serum, 4 mM l-glutamine, 100 U/mL penicillin, and 0.1 mg/mL streptomycin at 37 °C and 5% CO_2_ in a humidified incubator. After C2C12 myoblasts reached 70–80% confluence, differentiation was initiated by replacement of the culture medium with DM: DMEM containing 2% horse serum (Sigma-Aldrich Corp., St. Louis, MO, USA). The cells were additionally incubated until each experiment. C2C12, H9C2, and HEK293T cells were transfected with the indicated amount of siRNA or plasmid DNA using Lipofectamine RNAiMAX or Lipofectamine 2000 reagent (Invitrogen), respectively. We used 10 nM cocktails of 3 unique siRNA duplexes for silencing of mouse WNK1 (SR423399; OriGene), mouse SPAK (SMF27A-2154; Cosmo Bio USA Co., Carlsbad, CA, USA), mouse OSR1 (SR416746; OriGene), mouse FOXO4 (SR416251; OriGene), or mouse Cullin3 (SR419963; OriGene). Cells were lysed, 48 h after transfection, to analyze mRNA and protein expression levels as well as efficiencies of each gene silencing. To increase the transfection and knock-down efficiencies, we used a reverse transfection approach with 1.0–1.5 × 10^5^ cells in a 6-well plate 48 h before the cells reach 70–80% confluence. When differentiation was induced in the transfected C2C12 cells, the culture medium was replaced with DM 48 h after transfection. In the experiments using a WNK kinase inhibitor WNK463, DM was supplemented with either 0.1, 1, or 10 μM WNK463 (Selleck Chemicals, Houston, TX, USA)^34,47^ dissolved in DMSO (equally consisting of 0.1% DMSO in DM), or DMSO alone.

### Animals and treatment

All experiments were performed in accordance with the guidelines for animal research of Tokyo Medical and Dental University, and the protocol was approved by The Animal Care and Use Committee of Tokyo Medical and Dental University. Male C57BL/6 J mice (7 or 8 weeks old at arrival) were fed normal chow and water under standard lighting conditions (12-h:12-h light–dark cycle). On arrival, mice were randomly assigned to either the exercise or no exercise group. The exercise group performed voluntary wheel running for 6 weeks in a plastic cage (height, 140 mm; width, 215 mm; depth, 320 mm), and were given free access to a running wheel (wheel circumference, 140 mm; Melquest, Toyama, Japan)^8^. For the CKD model^29^, mice were also randomly assigned to either the CKD or control group. The CKD group mice were fed with a 0.25% adenine-containing diet (Sigma-Aldrich Corp.) for 4 weeks. WNK463 (10 mg/kg)^34^ or vehicle was administered by oral gavage. After 6 weeks of exercise, induction of CKD, or 6 h after treatment of WNK463, the mice were euthanized, blood samples were collected, and the quadriceps and kidneys were removed, respectively. Biochemical analysis of serum creatinine was performed as previously described^29^.

### Immunoblotting

C2C12 or H9C2 cells grown in a 6-well plate were lysed in RIPA buffer (50 mM Tris-HCl [pH 7.5], 150 mM NaCl, 0.5% sodium deoxycholate, 0.1% SDS, 1 mM EGTA, 1 mM EDTA, 1 mM sodium orthovanadate, 50 mM sodium fluoride, 1% Triton X-100, and Protease Inhibitor Cocktail [Roche Diagnostics, Basel, Switzerland]) for 30 min at 4 °C. The lysates were centrifuged at 12,000 × *g* for 5 min, and the supernatants were diluted with 2 × SDS sample buffer (Cosmo Bio) and denatured at 60 °C for 20 min. To separate nuclear/cytoplasmic extracts of C2C12 cells, nuclear and cytoplasmic extraction reagents (PIERCE, AZ, USA) were used according to the manufacturer’s protocol. Quadriceps muscle homogenates were lysed in a buffer as previously described^8^ and the lysates were centrifuged at 17,000 × *g* for 10 min. After centrifugation, the supernatants were diluted with 2 × SDS and denatured similarly.

The following primary antibodies were used: anti-WNK1 (A301-515A; BETHYL, Montgomery, TX, USA); anti-myogenin (F5D; Santa Cruz Biotechnology, Inc., Dallas, TX, USA; sc-12732); anti-MHC (A4.1025; Upstate Biotechnology Inc., Lake Placid, NY, USA); anti-phosphorylated Akt (S473; Cell Signaling; 4060); anti-Akt1/2/3 (Santa Cruz Biotechnology; sc-8312); anti-phosphorylated FOXO4 (T32 or S197; Cell Signaling; 2599 or 9471); anti-phosphorylated FOXO4 (S262; abcam, UK; ab126594); anti-FOXO4 (Cell Signaling; 9472); anti-FOXO1 (Cell Signaling; 2880); anti-FOXO3 (Cell Signaling; 2497); anti-Smad2/3 (Cell Signaling; 5678); anti-Histone H3 (Cell Signaling; 4499); anti-β-tubulin (Cell Signaling; 2128); anti-NF-κB p65 (Cell Signaling; 8242); anti-p38 MAPK (Cell Signaling; 8690); anti-phosphorylated SPAK^37^; anti-SPAK (Cell Signaling; 2281); anti-PPARδ (abcam; ab8937); anti-Actin (Cytoskeleton, Inc., Denver, CO, USA); anti-GAPDH (Santa Cruz Biotechnology; sc-32233); anti-FLAG (Sigma-Aldrich Corp.); and anti-Halo (Promega Corp., Madison, WI, USA). Alkaline phosphatase-conjugated anti-Immunoglobulin G antibodies (Promega Corp.) were used as secondary antibodies. The band densities of the proteins were quantified using ImageJ (National Institutes of Health, Bethesda, MD, USA). Immunoblots of WNK1 in mouse skeletal muscle, heart, WNK1-silenced C2C12 cells, or HEK293T cells after WNK1 overexpression were described in Fig. S6. The used antibody clearly showed the endogenous WNK1, its depletion by siRNA, or Halo-tagged human WNK1, suggesting the specific detection of this protein.

### Quantitative real-time reverse-transcription polymerase chain reaction

Total RNA was extracted with the Sepasol-RNA I Super G (Nacalai Tesque, Kyoto, Japan) from cultured cells or mouse tissues using the manufacturer’s protocol. cDNA was synthesized using ReverTra Ace® (Toyobo Co., Ltd., Osaka, Japan). Quantitative real-time RT-PCR was performed as previously described^48^. All reactions were performed in duplicate, and the relative mRNA expression level of each target gene was normalized with that of *β-actin* as an internal control. The used primers for RT-PCR are shown in Table S1.

### Immunofluorescence

C2C12 myotubes 96 h after the induction of differentiation were fixed in 3% paraformaldehyde in PBS for 15 min, blocked with 1% BSA in PBS for 30 min, and incubated in 0.2% Triton X-100 in PBS for 10 min. After three washes in PBS, the cells were treated with mouse anti-MHC antibody (A4.1025; Upstate Biotechnology Inc.) in PBS supplemented with 0.1% BSA for 4 h at room temperature. After three washes in PBS, the cells were incubated with 488 goat anti-mouse antibody (Molecular Probes, Eugene, OR, USA; A-11029; 1:200) with Hoechst 33342 for 1 h, washed three times in PBS. To analyze myotube diameter, we took pictures from each well and all obtained myotubes were included in the analysis. Each diameter of a single myotube was determined using ImageJ. We also analyzed the fusion index^8,49^, which represents the percentage of MHC-positive myotubes with ≥ two nuclei among the total myotubes within each field, using ImageJ.

### Statistics

Statistical significance was evaluated using the unpaired *t* test for two groups, or by one-way analysis of variance (ANOVA), followed by Bonferroni’s test for multiple groups. All data are presented as the mean ± standard error of the mean, and *P* < 0.05 was considered statistically significant.

### Data availability

All data generated or analysed during this study are included in this published article (and its Supplementary Information files).

## Electronic supplementary material

Supplementary Information
