# Supplementary material for: WNK1 regulates skeletal muscle cell hypertrophy by modulating the nuclear localization and transcriptional activity of FOXO4
Source: Sci Rep. 2018 Jun 14;8:9101. doi: 10.1038/s41598-018-27414-0 (PMC6002401; doi:10.1038/s41598-018-27414-0)
Supplement: Supplementary file 1 — Supplementary Information [file 41598_2018_27414_MOESM1_ESM.pdf]

# Supplementary Information

## WNK1 regulates skeletal muscle cell hypertrophy by modulating the nuclear localization and transcriptional activity of FOXO4

Shintaro Mandai[1], Takayasu Mori[1], Naohiro Nomura[1], Taisuke Furusho[1], Yohei Arai[1], Hiroaki Kikuchi[1], Emi Sasaki[1], Eisei Sohara[1], Tatemitsu Rai[1], Shinichi Uchida[1]*

[1]Department of Nephrology, Graduate School of Medical and Dental Sciences, Tokyo Medical and Dental University, 1-5-45 Yushima, Bunkyo, Tokyo 113-8519, Japan.

*Corresponding author: Shinichi Uchida

Department of Nephrology, Graduate School of Medical and Dental Sciences, Tokyo Medical and Dental University, 1-5-45 Yushima, Bunkyo, Tokyo 113-8519, Japan

Tel: +81-3-5803-5214; Fax: +81-3-5803-5215; E-mail: suchida.kid@tmd.ac.jp

**Supplementary Methods**

***Plasmids***

Plasmids for Halo-tagged human with-no-lysine (K) 1 (WNK1) and 3xFLAG-tagged human kelch like family member 3 (KLHL3) were previously described[38,44,45]. Human FOXO4 complementary DNA (cDNA) was isolated by RT-PCR using human kidney mRNA, and the cDNA was cloned into 3xFLAG-CMV10 vector (Sigma-Aldrich Corp., St. Louis, MO, USA). Human WNK1 plasmid was also used in the overexpression experiment in mouse C2C12 cells, because of the very high sequence homology between human and mouse WNK1 proteins particularly in their functional domains[49].

***Phos-tag SDS-PAGE***

HEK293T cells in a 6-well plate, 48 h after transfection of mouse WNK1 siRNA and 24 h after that of 1 μg human FOXO4 plasmid, were lysed with lysis buffer [50 mM Tris-HCl (pH 7.5), 150 mM NaCl, 1 mM EGTA, 1 mM EDTA, 1 mM sodium orthovanadate, 50 mM sodium fluoride, 1% Triton X-100, 0.27 M sucrose, 1 mM DTT, and protease inhibitor cocktail (Roche Diagnostics)]. The cell lysates were centrifuged and denatured as described in the Methods. Western blotting and Phos-tag western blotting[33] were performed to assess phosphorylated FLAG-FOXO4, using Phos-tag™ Acrylamide (50 μmol/L; Wako Pure Chemical Industries, Ltd., Osaka, Japan).

***Immunoprecipitation***

HEK293T or C2C12 cells transfected with 1 μg plasmids were lysed in a buffer (50 mM Tris-HCl [pH 7.5], 150 mM NaCl, 1% Nonidet P-40, 1 mM sodium orthovanadate, 50 mM sodium fluoride, and protease inhibitor cocktail) for 30 min at 4°C. After centrifugation at 15,000 *g* for 10 min, the supernatants were used for immunoprecipitation with anti-FLAG M2 beads (Sigma-Aldrich Corp.) for 2 hr at 4°C. The precipitants were washed with the lysis buffer and the immunoprecipitates were eluted in SDS sample buffer after boiling for 10 min.

**Supplementary Figure 1.** Effect of WNK1 gene silencing on relative expression levels of MAFbx and MuRF1 mRNA in H9C2 rat myocytes.

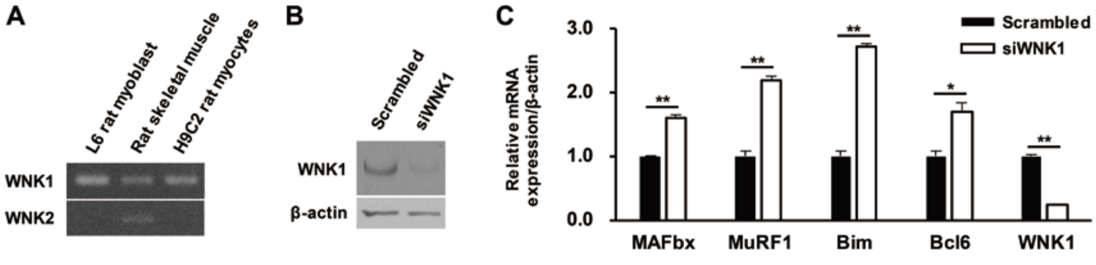


**A,** Reverse transcription polymerase chain reaction for WNK1 and WNK2 in rat skeletal muscle, L6 myoblasts, and H9C2 myocytes. **B,** Efficiency of small interfering RNA-mediated silencing of WNK1 at protein level in H9C2 rat myocytes revealed by immunoblots. **C,** Quantification of MAFbx, MuRF1, Bim, Bcl6, and WNK1 by real-time polymerase chain reaction analysis after WNK1 silecing (*n* = 3 per experimental group). Values are presented as the mean ± standard error of the mean. *$P$ < 0.05; **$P$ < 0.01 versus the control cells. WNK, with-no-lysine (K); MAFbx, muscle atrophy F-box; MuRF1, Muscle RING-finger protein-1. Full-length blots are presented in Supplementary Figure 14.

**Supplementary Figure 2.** WNK1 overexpression did not modulate myotube diameter and atrogene expression in C2C12 cells.

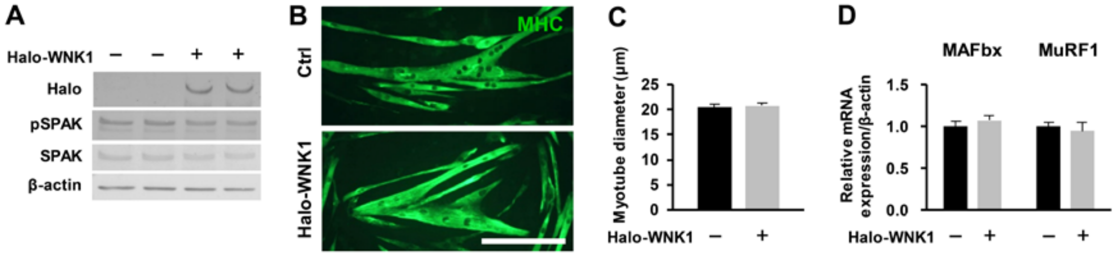


**A,** Immunoblots showing that WNK1 overexpression did not increase phosphorylation of SPAK in C2C12 cells. **B,** Immunofluorescence with a myosin heavy chain (MHC) antibody in C2C12 cells that were transfected with Halo-tagged human WNK1 and incubated in differentiation medium for 4 days. Scale bar, 200 μm. **C,** The mean myotube diameter was not altered by WNK1 overexpression ($n$ = 5 per experimental group). **D,** Quantification of MAFbx and MuRF1 in C2C12 cells that overexpress WNK1 by real-time polymerase chain reaction analysis ($n$ = 7 per experimental group). mRNA levels were normalized against those of *β-actin*. Values are presented as the mean ± standard error of the mean. WNK, with-no-lysine (K); MHC, myosin heavy chain; MAFbx, muscle atrophy F-box; MuRF1, Muscle RING-finger protein-1; SPAK, STE20/SPS1-related proline/alanine-rich kinase. Full-length blots are presented in Supplementary Figure 15.

**Supplementary Figure 3.** Protein abundances of NF-κB p65 and p38 MAPK in nuclear and cytoplasmic fractions of WNK1-silenced C2C12 cells.

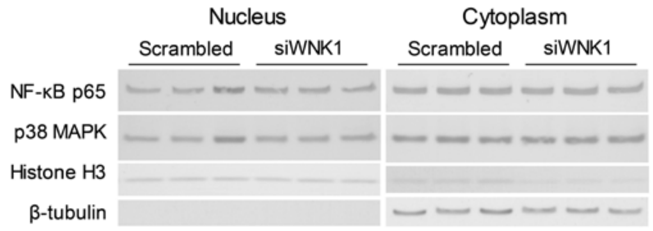


Immunoblots of NF-κB p65 and p38 MAPK after separating nuclear (upper)/cytoplasmic (lower) extracts in C2C12 cells treated with WNK1-targeted siRNA showed that their nuclear and cytoplasmic localization was not altered. WNK, with-no-lysine (K); NF-κB, Nuclear factor-κB; MAPK, mitogen-activated protein kinase. Full-length blots are presented in Supplementary Figure 16.

**Supplementary Figure 4.** A WNK kinase inhibitor WNK463 increased atrogene expression in mouse skeletal muscle and C2C12 cells.

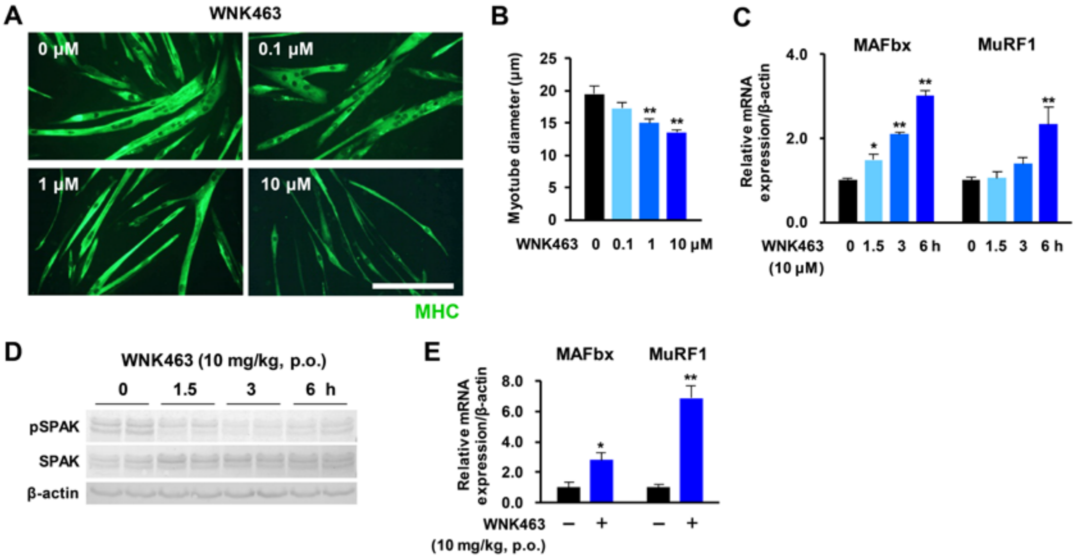


**A,** Immunofluorescence with a myosin heavy chain (MHC) antibody in C2C12 cells that were incubated in differentiation medium with either 0.1, 1, or 10 μM WNK463, or DMSO alone for 4 days. Scale bar, 200 μm. **B,** WNK463 induced a dose-dependent decrease in the C2C12 myotube diameter ($n$ = 5 per experimental group). **C,** MAFbx and MurRF1 mRNA expression levels were time-dependently increased by 10 μM WNK463 ($n$ = 4 per experimental group). **D,** Immunoblots of total (t) and phosphorylated (p) SPAK in the whole kidney lysates from wild type mice at 0, 1.5, 3, and 6 h after treatment with WNK463 (10 mg/kg, p.o.), demonstrating the sufficient suppression of WNK kinase activity *in vivo*. **E,** Atrogene transcription was induced in mouse quadriceps at 6 h after administration of WNK463 (10 mg/kg, p.o.) ($n$ = 6 per experimental group). mRNA levels were normalized against those of *β-actin*. Values are presented as the mean ± standard error of the mean. *$P$ < 0.05; **$P$ < 0.01 versus the control group. WNK, with-no-lysine (K); MHC, myosin heavy chain; MAFbx, muscle atrophy F-box; MuRF1, Muscle RING-finger protein-1; SPAK, STE20/SPS1-related proline/alanine-rich kinase. Full-length blots are presented in Supplementary Figure 17.

**Supplementary Figure 5.** Relative expression levels of WNK1 mRNA in skeletal muscle of hypertrophy or atrophy model mice.

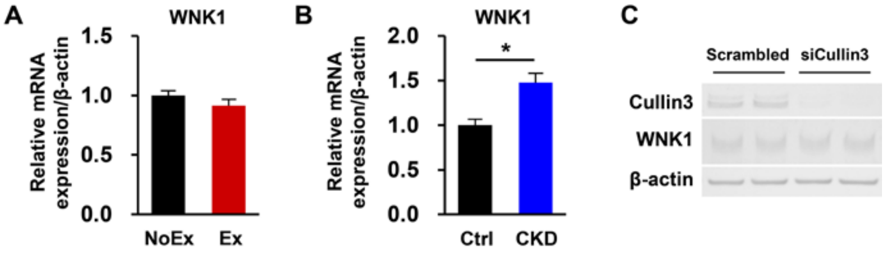


**A–B,** Quantification of WNK1 mRNA in mouse quadriceps by real-time polymerase chain reaction analysis after 6 weeks of voluntary wheel running exercise **(A)** ($n$ = 5 per experimental group) or induction of chronic kidney disease (CKD) **(B)** ($n$ = 3 per experimental group). The modulation of WNK1 mRNA expression was not responsible for the altered WNK1 protein levels in either mouse muscle hypertrophy or atrophy model. The increases of WNK1 mRNA by CKD might reflect a compensatory response to the marked decreases in WNK1 protein levels. mRNA levels were normalized against those of *β-actin*. Values are presented as the mean ± standard error of the mean. $^*P < 0.05$ versus the control mice. **C,** Immunoblots of WNK1 in C2C12 cells after small interfering RNA-mediated silencing of Cullin3, showing that Cullin3 does not modulate WNK1 protein expression in muscle cells. Ex, exercise; NoEx, no exercise; Ctrl, control; WNK, with-no-lysine (K). Full-length blots are presented in Supplementary Figure 18.

**Supplementary Figure 6.** Immunoblots of WNK1 in mouse tissues, C2C12 or HEK293T cells after WNK1 silencing or overexpression.

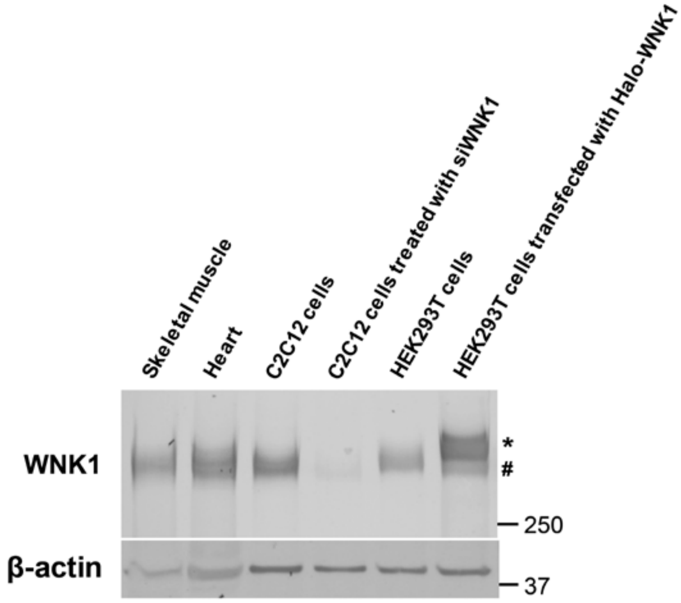


Immunoblots of WNK1 in C2C12 cells, HEK293T cells, skeletal muscle and heart tissues from a wild-type mouse (10 µg or 40 µg per lane for each cell or tissue lysate, respectively). The asterisk (*) and the hash (#) show Halo-tagged human WNK1 and endogenous WNK1, respectively. WNK, with-no-lysine (K). Full-length blots are presented in Supplementary Figure 19.

**Supplementary Figure 7.**

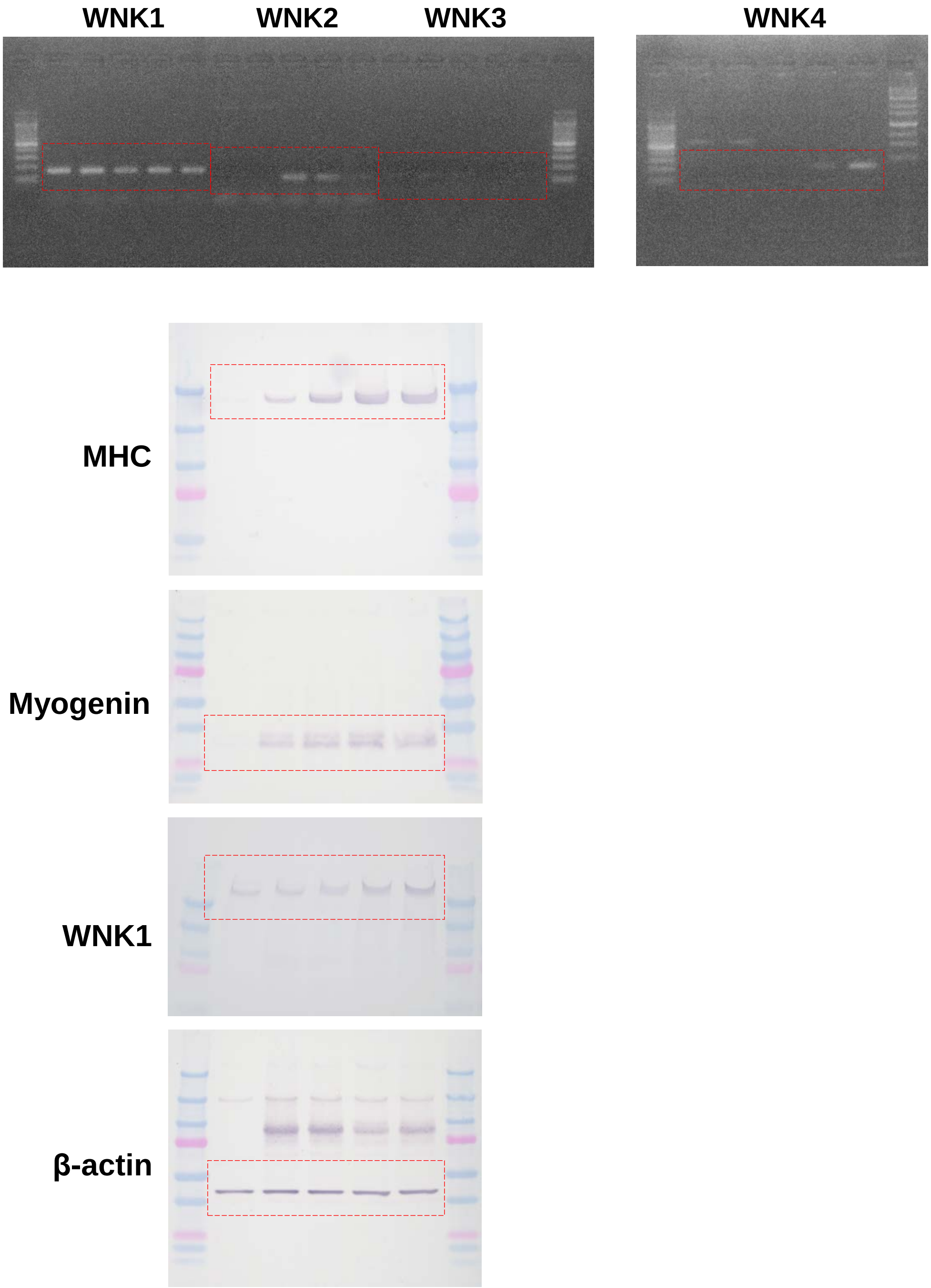


Original western blots or whole gels for the images shown in Figure 1. The cropped images are highlighted in the red lines.

**Supplementary Figure 8.**

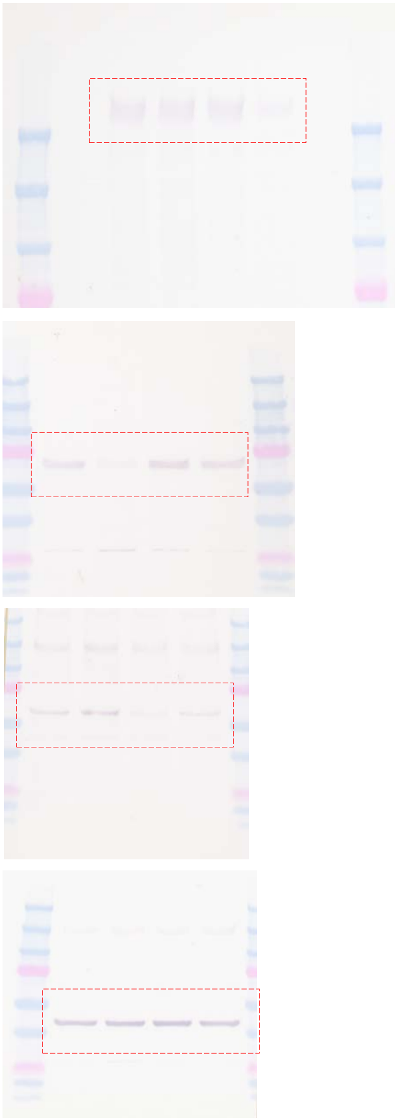

WNK1

SPAK

OSR1

β-actin

Original western blots for the images shown in Figure 2. The cropped images are highlighted in the red lines.

**Supplementary Figure 9.**

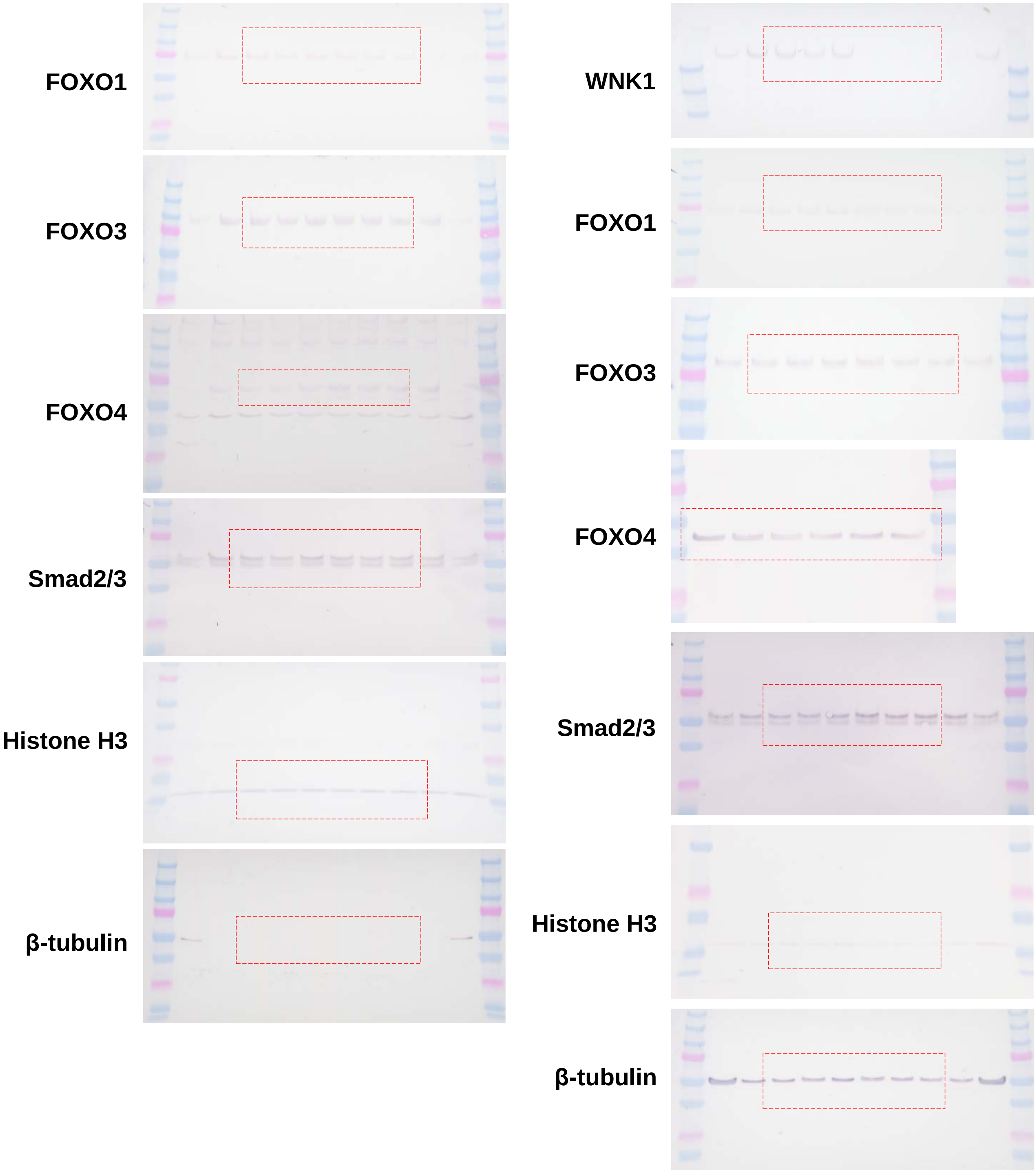


Original western blots for the images shown in Figure 3. The cropped images are highlighted in the red lines.

**Supplementary Figure 10.**

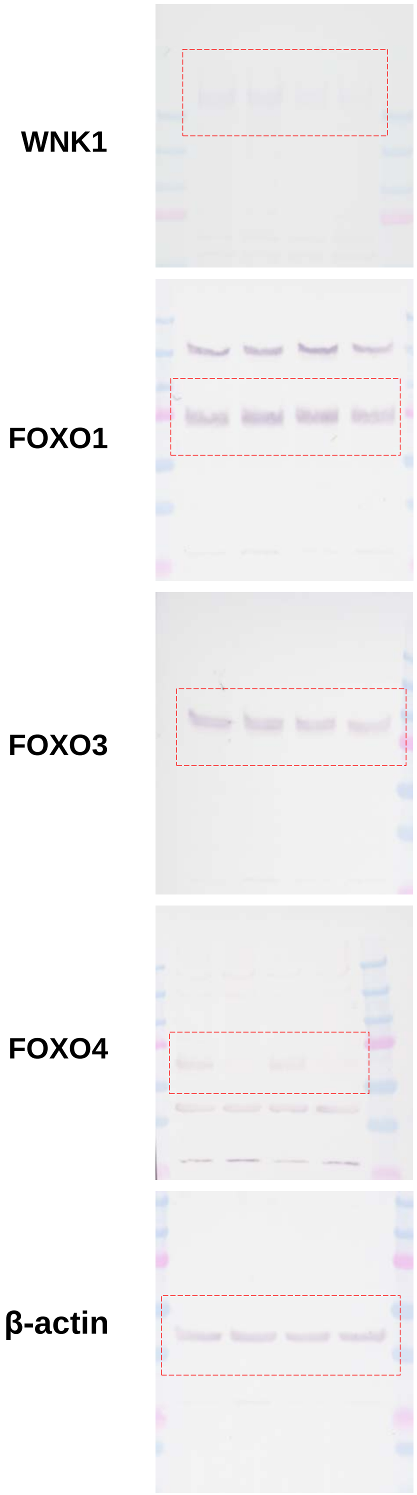


Original western blots for the images shown in Figure 4. The cropped images are highlighted in the red lines.

**Supplementary Figure 11.**

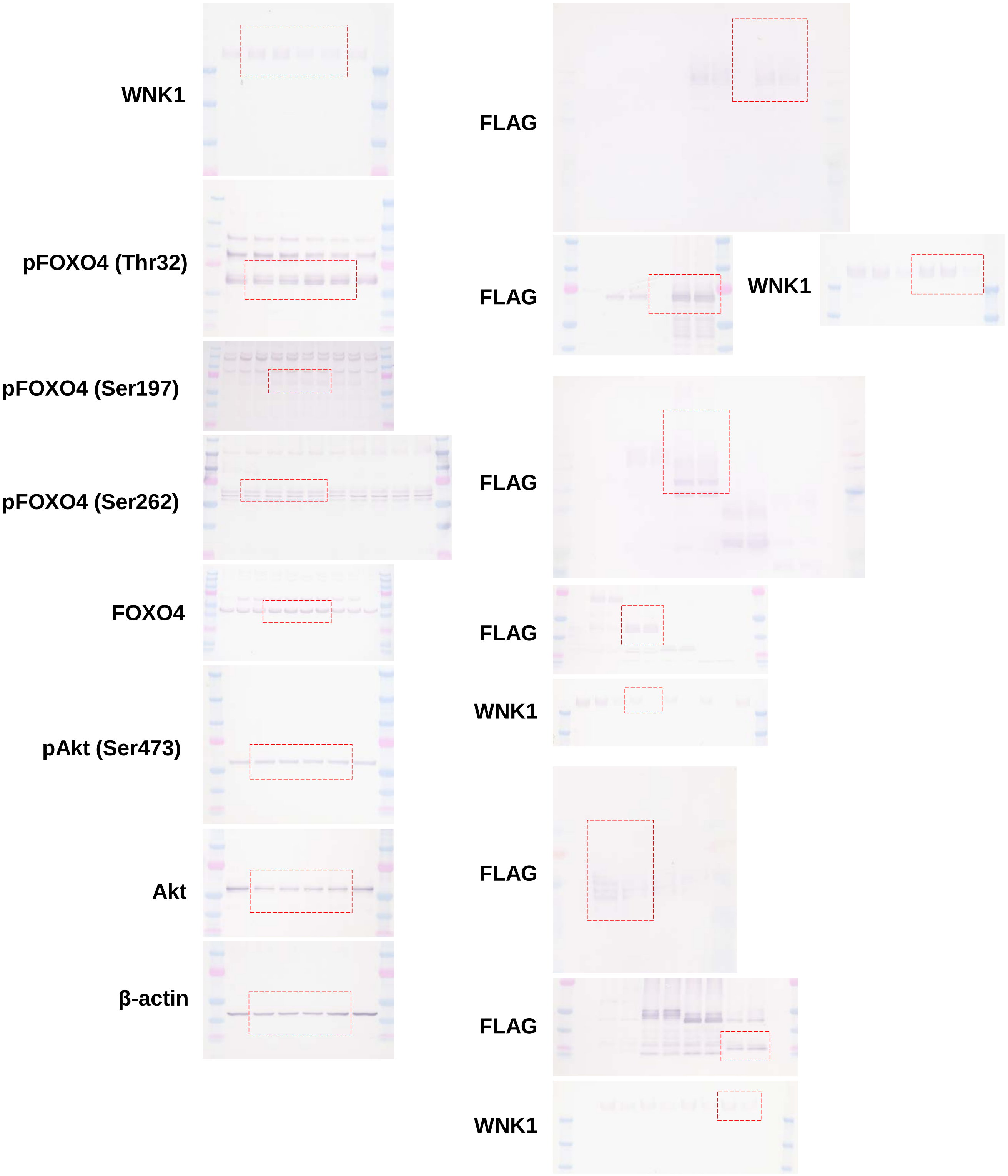


Original western blots for the images shown in Figure 5. The cropped images are highlighted in the red lines.

**Supplementary Figure 12.**

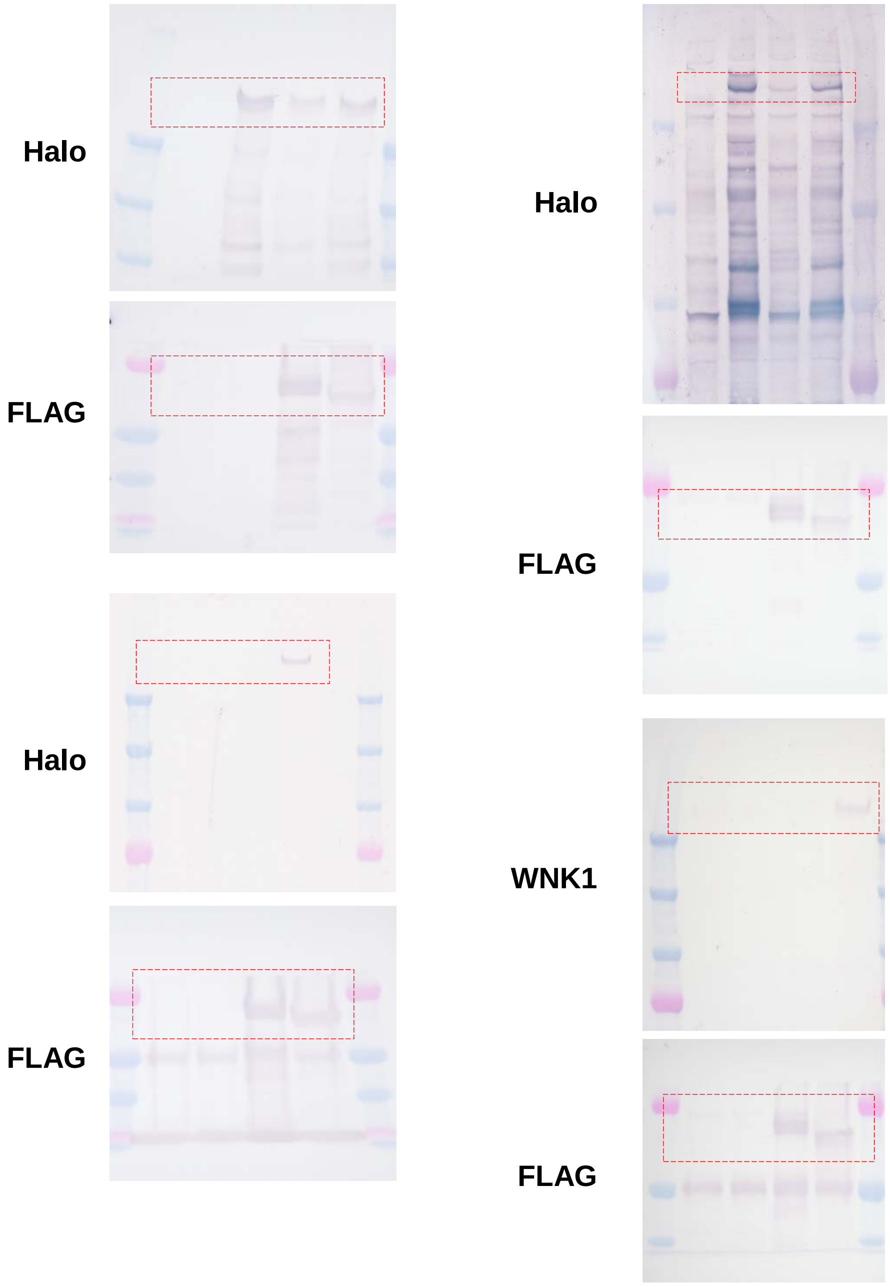


Original western blots for the images shown in Figure 6. The cropped images are highlighted in the red lines.

**Supplementary Figure 13.**

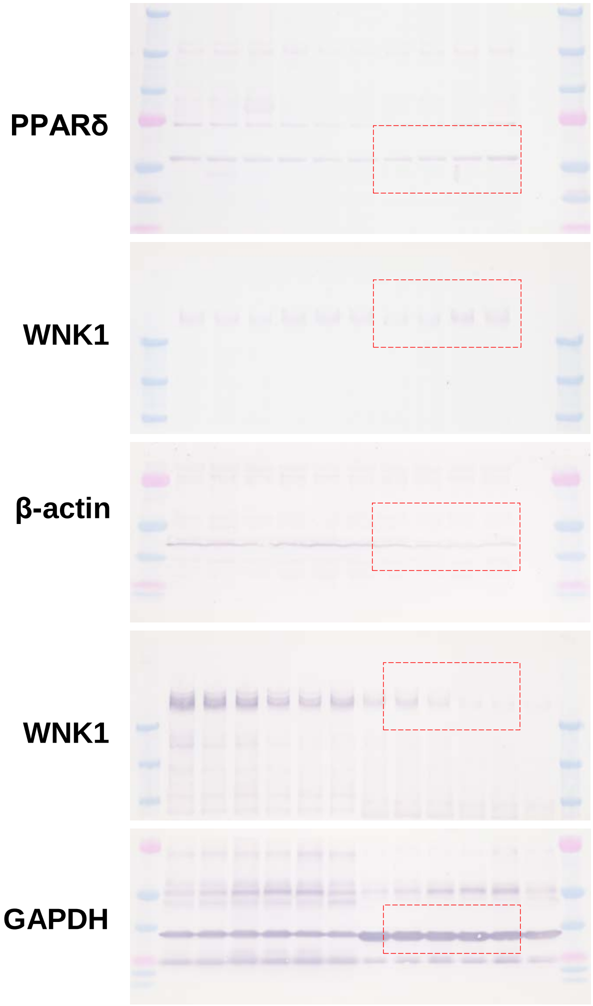


Original western blots for the images shown in Figure 7. The cropped images are highlighted in the red lines.

**Supplementary Figure 14.**

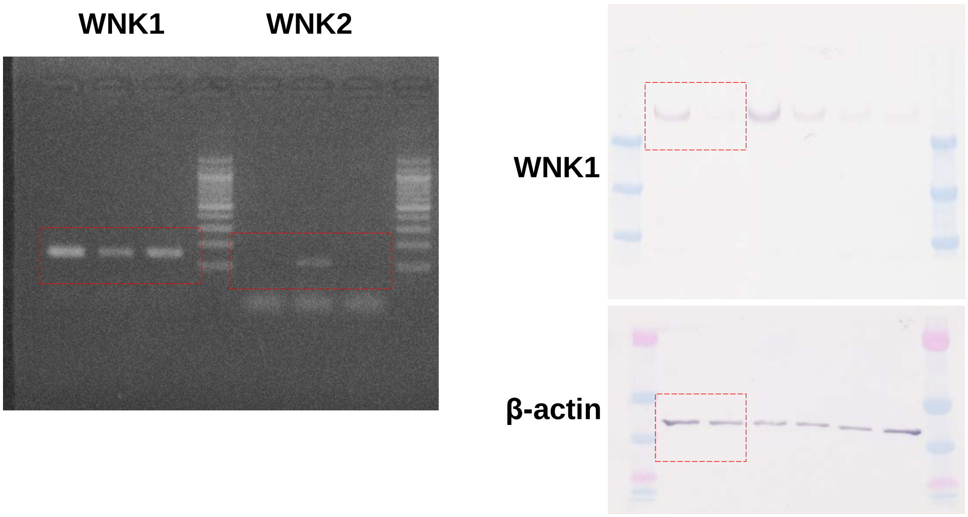


Original western blots or whole gels for the images shown in Supplementary Figure 1. The cropped images are highlighted in the red lines.

**Supplementary Figure 15.**

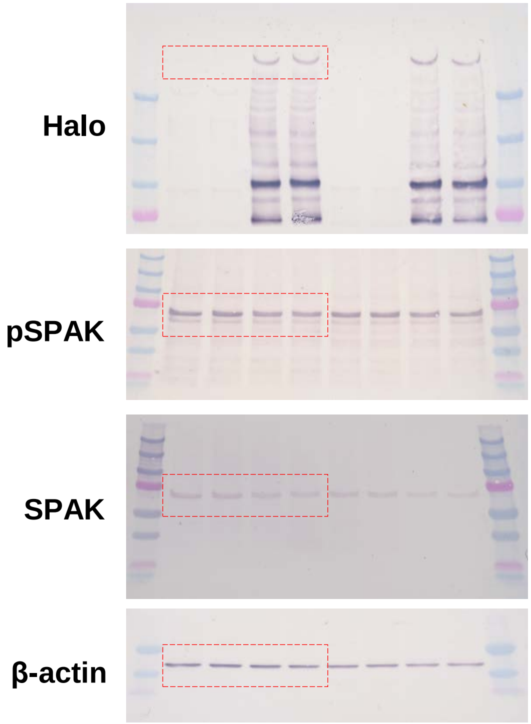


Original western blots for the images shown in Supplementary Figure 2. The cropped images are highlighted in the red lines.

**Supplementary Figure 16.**

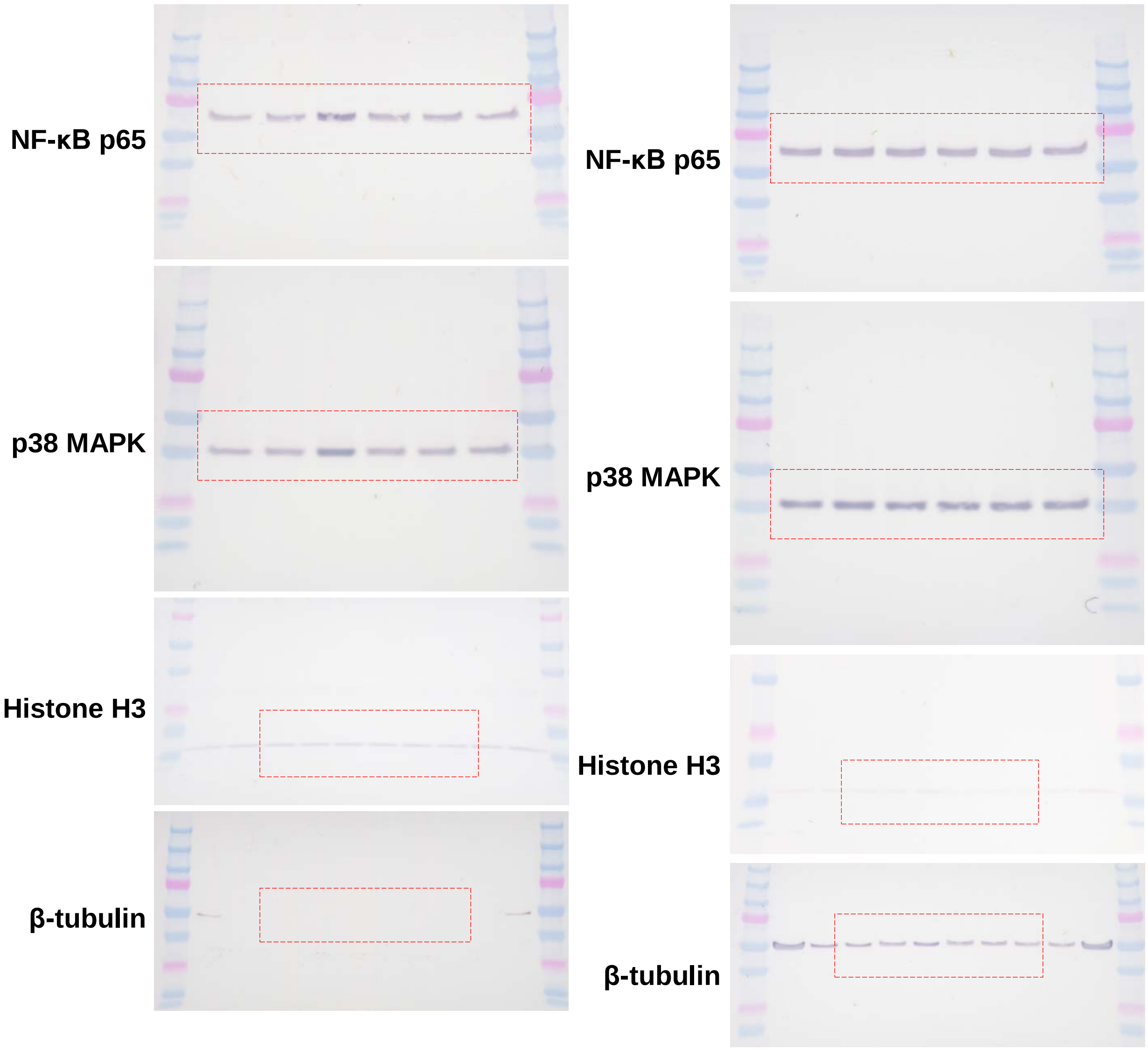


Original western blots for the images shown in Supplementary Figure 3. The cropped images are highlighted in the red lines.

**Supplementary Figure 17.**

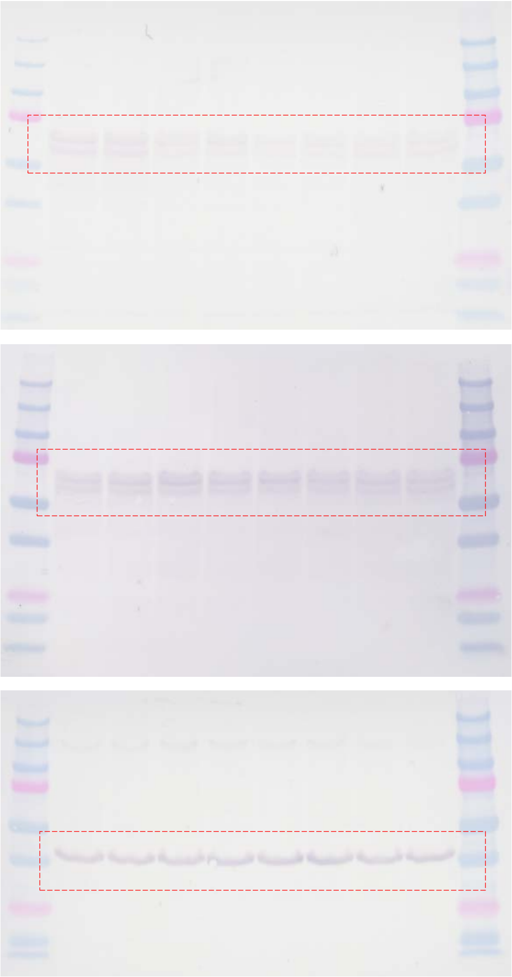

pSPAK

SPAK

β-actin

Original western blots for the images shown in Supplementary Figure 4. The cropped images are highlighted in the red lines.

**Supplementary Figure 18.**

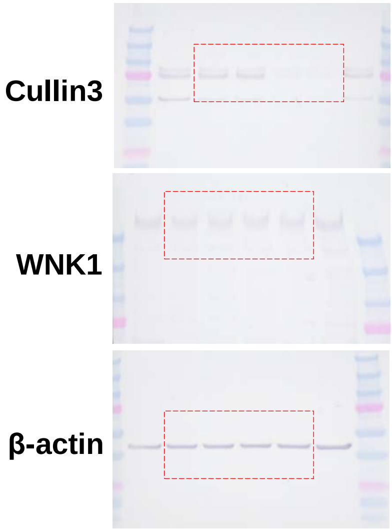


Original western blots for the images shown in Supplementary Figure 5. The cropped images are highlighted in the red lines.

**Supplementary Figure 18.**

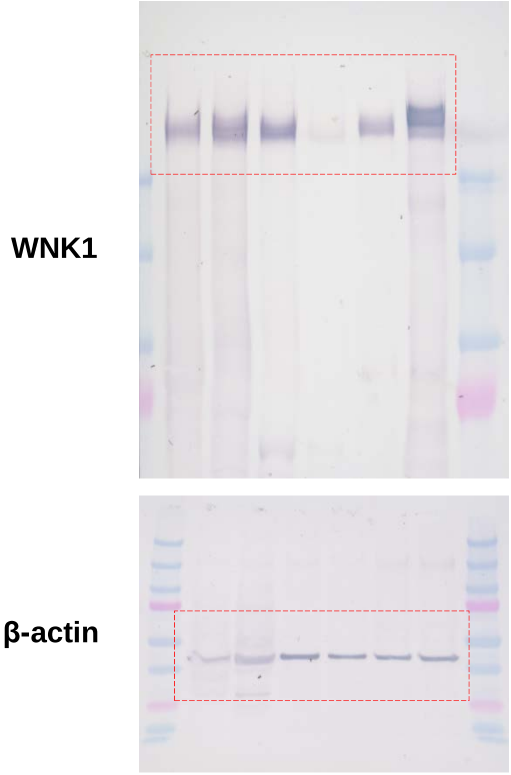


Original western blots for the images shown in Supplementary Figure 6. The cropped images are highlighted in the red lines.

**Supplementary Table 1.** Primer sequences used for reverse-transcription polymerase chain reaction

| Genes | Species | Amplicon size (bp) | Forward primer | Reverse primer |
|---|---|---|---|---|
| *Actb (β-actin)* | Mouse | 137 | CTCTGGCTCCTAGCACCATG | GTACTCCTGCTTGCTGATCC |
| *Bcl2l11 (Bim)* | Mouse | 122 | GGCCAAGCAACCTTCTGATG | CTTGCGGTTCTGTCTGTAGG |
| *Bcl2l11 (Bim)* | Rat | 122 | GGCCAAGCAACCTTCTGATG | CTTGCGATTCTGTCTGTAGG |
| *Bcl6* | Mouse | 142 | TCGTGGTGAGCCGTGAGCAG | AACCCCTCAGGGCTGATTTC |
| *Bcl6* | Rat | 142 | TCGTGGTGAGCCGTGAGCAG | AACCCCTCAGGGCTGATCTC |
| *Fbxo32 (MAFbx)* | Mouse | 223 | AAGGCTGTTGGAGCTGATAGCA | CACCCACATGTTAATGTTGCCC |
| *Fbxo32 (MAFbx)* | Rat | 223 | AAGGCTGTTGGAGCTGATAGCA | CACCCACATGTTGATGTTGC |
| *Pmaip1 (Noxa)* | Mouse | 116 | CTGTGGTTCTGGCGCAGATG | TCCAATCCTCCGGAGTTGAG |
| *Trim63 (MuRF1)* | Mouse | 143 | TGCCTGGAGATGTTTACCAAGC | AAACGACCTCCAGACATGGACA |
| *Trim63 (MuRF1)* | Rat | 141 | CCTTGAGATGTTTACCAAGC | AAACGACCTCCAGACATGGACA |
| *WNK1* | Mouse | 168 | CTACAAAGGTCTGGACACCG | ACTGTGGATTCCCAGGAATC |
| *WNK1* | Rat | 150 | ACTGTGGAAGTCGCCTGGTG | CCCTTTTACTGTGGATTCCC |
| *WNK2* | Mouse | 113 | TGATGACCTCGGGTACACTG | TGTATGCAGGAACAGCAGGC |
| *WNK2* | Rat | 113 | TGATGACCTCAGGGACATTG | TGTGTGCAGAAACAACAGGC |
| *WNK3* | Mouse | 129 | TGTTGCAACTTCCCCTAGTG | TCCTGTAGCTCACACCAAGC |
| *WNK4* | Mouse | 215 | TGTCTATTCCACGGTCTGGC | GTTGTGAGTCTGCAGCTGAC |

*Bcl2l11, BCL2-like 11; Bcl6, B cell leukemia/lymphoma 6; Fbxo32, F-box protein 32; Pmaip1, phorbol-12-myristate-13-acetate-induced protein 1; Trim63, tripartite motif-containing 63; MAFbx, muscle atrophy F-box; MuRF1, Muscle RING-finger protein-1; MUSA1, muscle ubiquitin ligase of the SCF complex in atrophy-1; WNK, with-no-lysine (K).*
